# Future Range Shifts in Major Maize Insect Pests Suggest Their Increasing Impacts on Global Maize Production

**DOI:** 10.3390/insects16060568

**Published:** 2025-05-28

**Authors:** Qiance Wei, Xueyou Zhang, Fang Yang, Sixi Duan, Zejian Fan, Peixiao Nie, Zhihong Chen, Jianmeng Feng

**Affiliations:** 1 College of Agriculture and Biological Science, Dali University, Dali 671003, China; 20242011100040@stu.dali.edu.cn (Q.W.); zhangxueyou0604@163.com (X.Z.);; 2 Research Center for Agro-Ecology in Erhai Lake Watershed, Dali University, Dali 671003, China; 3 Cangshan Forest Ecosystem Observation and Research Station of Yunnan Province, Dali University, Dali 671003, China; 4 Inspection Institution of Agricultural Environment-Resource and Agricultural Products in Dali, Dali 671001, China

**Keywords:** climate, future range shifts, impacts, maize production, maize pests

## Abstract

Maize is one of the three staple grains, and its global demand has risen sharply in recent decades. However, insect pests are causing significant production losses. Despite this, few studies have yet investigated future range shifts in major insect pests affecting maize. Here, we used a unified framework to build 24 multi-algorithm models to forecast their future range shifts under future climate change scenarios (SSP126 and SSP585, representing optimistic and pessimistic scenarios, respectively). Habitat suitability was projected to increase in most regions. Significant range expansions were identified for all of them, with future climate changes being the primary driver for most. High-range overlaps were predominantly observed in the USA, Mexico, and other regions. We also identified species showing the largest ranges and range shifts, suggesting the priority species in our strategies against their impacts on maize. The relative roles of climate and crop availability in the range dynamics of major insect pests affecting maize could be, to a certain extent, determined by whether they are monophagous on crop hosts or not. High-range overlap in key maize-producing regions highlights the substantial threat they pose to global maize production. Therefore, mitigating future climate changes could be a crucial strategy to reduce their impacts on future maize production.

## Simple Summary

This study built 24 multi-algorithm SDMs to calibrate range shifts in major insect pest species affecting maize and to assess their controlling factors within a unified framework. We observed overall increases in habitat suitability in most regions. Significant range expansions were identified for all of them, with future climate changes being the primary driver for most. The relative influence of climate and crop availability on range dynamics could be, to a certain extent, determined by whether they are monophagous on crop hosts or not. Future range expansions of major maize pests highlight their potential for increasing impacts on global maize production.

## 1. Introduction

Maize is one of the three staple grains. Although over half the maize produced globally is consumed by livestock, the demand for maize grain has risen sharply in recent decades [1]. This increase may be attributed to its diverse uses, such as oil in foods, corn sugar in beverages, flour in bakery products, and cornstarch in industrial applications [2]. The high demand for maize has, to some extent, outpaced supply [3]. Besides enhancing maize production, reducing losses caused by pest impacts is critical to ensuring global maize supply, as insect pest damage and pest control costs represent the largest resource allocations in maize production worldwide [4,5,6,7]. However, the threats posed by insect pests to global maize production are projected to intensify in the future [8,9,10]. Therefore, mitigating the future impacts of pests on maize production is imperative. In this study, we hypothesized that understanding the future range shifts of major maize pests could provide valuable insights for developing effective strategies to minimize their impacts on global maize production.

Climate change is one of the most frequently studied factors in projections of future range shifts for insect pests affecting maize. For example, Matsukura et al. observed climate-change-induced expansions in the potential range of *Cicadulina bipunctata* (Melichar) (Homoptera: Cicadellidae), a major maize pest in Japan [11]. Similarly, Senay et al. projected range expansions for *Spodoptera frugiperda* (JE Smith) (Lepidoptera, Noctuidae), another significant maize pest, under climate-change scenarios in the future [12]. They argued that climate change could increase the threats posed by insect pests to maize production in the future. However, Santana Jr et al. projected climate-change-induced range contractions for *Dalbulus maidis* (DeLong) (Hemiptera: Cicadellidae) in the future, and therefore climate change might decrease the impacts of some insect pests on future maize production [13]. Thus, the roles of future climate change in the range shift of maize pest insects remain a topic of debate, and much more attention is deserved to elucidate the role of climate change in shaping the future distributions of these pests.

For most insect pests, crops serve as one of their primary food resources, in addition to climatic conditions, crop availability could exert important influences on the range shifts of insect pests on maize. Mengesha et al. argued that crop availability strongly influenced the potential ranges of *S. frugiperda* in southern Ethiopia [14]. Liu et al. similarly found that crop availability was an important factor in the global range shifts of a maize insect pest [15]. Nevertheless, to our best knowledge, most study cases on the range shifts of maize insect pests have focused their attention on the impacts of climate-change-induced range shifts in one or a few insect pests on maize [6,16,17], with limited attention given to the relative strength of climate and crop availability for the range shift in most major maize insect pests. Therefore, further investigations are urgently needed in this area.

Although topographical factors are closely associated with nutrient storage and the spatial distribution of water and energy [18,19,20,21], few studies, to our knowledge, have examined the effects of topographical patterns on the range shifts of insect pests on maize. However, this does not necessarily imply that topographical factors have no influence on their range shifts. For example, steeper slopes can lead to higher losses of dissolved total nitrogen in maize fields, resulting in lower maize yields [22]. This suggests that flat regions may provide more favorable habitats for maize, which explains why most maize farms are located in flat areas [23,24]. Consequently, insect pests on maize are more likely to be found in flat regions, as these areas facilitate easier access to host plants. Therefore, the influence of topographical variables on the range shifts of maize insect pests should not be overlooked.

Finally, we observed that several studies have investigated the range alterations in insect pests affecting maize under future scenarios. For example, Jin et al. forecasted the global range expansions of *Diabrotica virgifera virgifera* (LeConte) (Coleoptera: Chrysomelidae), while Paudel Timilsena et al. examined the future range alterations in *S. frugiperda* in Africa [17,25]. Nevertheless, to our best knowledge, most of them have focused primarily on *S. frugiperda* and a few other insect pests on maize. Furthermore, none have comprehensively investigated the future range shifts of all major insect pests on maize using a unified framework that could provide critical insights for strategy development. Such information is crucial for assessing the relative risks of different insect pests on maize and identifying hotspots of their potential impacts.

Here, we utilized climatic, crop availability, and topographical variables to forecast the global range alterations of major insect pests on maize through a unified approach. Our objectives were to investigate their overlapping ranges, identify hotspots of range expansion, evaluate their relative risks, and discuss their implications for global maize production under future scenarios. We also hypothesized that the relative influence of climate, crop availability, and topographical factors on the future range alterations in these pests may vary between species. Our findings provide novel and essential perceptions for building effective strategies to mitigate the impacts of pest insects on future global maize production.

## 2. Materials and Methods

### 2.1. The Study Area

With a reference to the Land-Use Harmonization online dataset (LUH2, https://luh.umd.edu/, accessed on 21 January 2024), we detected regions that could not be cropland under current and future scenarios. Then, we have excluded all of them from our study area, which, to a certain extent, could remove the regions where maize cultivation is biophysically impossible.

### 2.2. Occurrence Records

**Figure 1:**
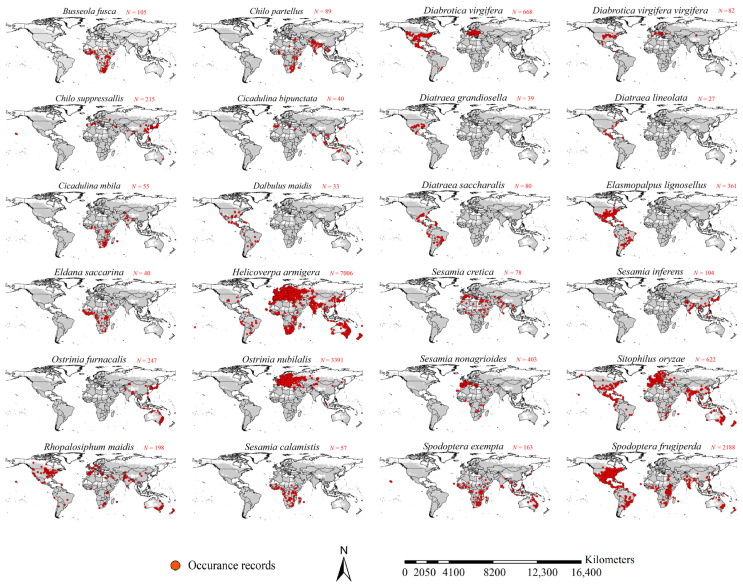
Occurrence points of the 24 major maize insect pest species.

Although numerous studies have reported or reviewed insect pests on maize [26,27,28,29,30,31,32], most of them paid attention to the maize insect pests in their local regions. Our study retrieved major insect pest species on maize from a publication by Steffey et al. [33], which comprehensively reviewed major maize insect pests in the world. Their occurrence records were then retrieved from literature survey and online data sources, including the Global Biodiversity Information Facility (GBIF, https://www.gbif.org, accessed on 21 February 2024), the European and Mediterranean Plant Protection Organization (EPPO, https://gd.eppo.int, accessed on 22 February 2024), and the Center for Agriculture and Bioscience International (CABI, https://www.cabi.org/ISC, accessed on 22 February 2024). In total, 87,073 global occurrences of the major insect pest species on maize were retrieved. A dataset of occurrence records for each species was also compiled. Following the approach described by Nie et al., occurrences with geographic coordinate uncertainties of ≥5 km were excluded from these datasets [34]. Additionally, we removed the occurrences which were outside of the study area, i.e., global cropland. In consideration of sample bias in our occurrence records, especially in those from GBIF [35], we used a spatial rarefication method recommended by Cao and Feng [36], i.e., retaining only one record within each 5 × 5 km grid. Additionally, to further reduce the influences of sample bias, we used the SDM toolbox [37] to generate sample bias maps for each species which were then applied to weigh the occurrence records. We removed the insect pests that had less than 30 occurrences after the spatial rarefication. This process resulted in 24 major insect pests on maize with a total of 16,291 records, which were used to construct the final dataset of occurrence records for each species (Figure 1 and Table S1).

### 2.3. Selecting Predictors

Three groups of 29 predictors were compiled to investigate the global potential ranges of 24 major insect pest species on maize (Table S2). These 29 predictors included climate (19 predictors), crop availability (7), and topographical patterns (3) (Table S2). Current climatic predictors represented climate conditions from 1990 to 2020 on a global scale. To obtain these predictors, spatial grid layers of monthly-averaged temperature and precipitation were retrieved from the Climate Research Division (CRU, https://crudata.uea.ac.uk/, accessed on 27 March 2024) at a spatial resolution of 2.5-arc-minute. The ‘Biovarcs’ R package was then utilized to generate current climatic factors, which were consistent with the 19 bioclimatic predictors in Worldclim [38]. The future climatic predictors for 2100 were acquired from Worldclim (www.worldclim.org, accessed on 23 February 2024). These predictors were generated using two mutually complementary and robust global climate models (GCMs): FIO-ESM-2 (FIO) and MPI-ESM-HR (MPI) [39]. Two future scenarios were examined, namely, the shared socio-economic pathways (SSP) 126 and 585, representing optimistic and pessimistic scenarios, respectively. Therefore, five datasets of climate variables were obtained: datasets under the current scenario, datasets under the SSP126 scenario generated through FIO (FIO126), datasets under the SSP585 scenario generated through FIO (FIO585), datasets under the SSP126 scenario generated through MPI (MPI126), and datasets under the SSP585 scenario generated through MPI (MPI585). Eight predictors of crop availability were compiled: Fractions of maize, fractions of C3 perennial crop, C3 annual crop, C4 perennial crop, C4 annual crop, C3 crop, C4 crop, and total crop. Specifically, fractions of maize at spatial resolution of 30 s were downloaded from Earthstst (http://www.earthstat.org/, accessed on 20 January 2024), while other predictors of crop availability were compiled from the Land-Use Harmonization online dataset (LUH2, https://luh.umd.edu/, accessed on 21 January 2024), with a spatial resolution of 0.25-arc-degree. Of note, fractions of maize remain constant under current and future scenarios due to the lack of datasets for maize fractions in the future. The fractions of the C3 crop were calculated as the sum of C3 annual and C3 perennial crops, while those of the C4 crop were the sum of C4 annual and C4 perennial crops. The fractions of the total crop represented the combined fractions of C3 and C4 crops (Table S2). These predictors were resampled to a spatial resolution of 2.5-arc-minute. Furthermore, these three datasets were included: the current crop availability variable dataset, that under SSP126, and that under SSP585 in 2100. As one of the topographical factors, elevation with 0.5 arc-minute-spatial resolution was also retrieved from the WorldClim database (www.worldclim.org, accessed on 21 December 2023). Slope and aspect were calculated through the elevation data and subsequently resampled to a 2.5-arc-minute resolution (Table S2). Finally, it was noted that the potential ranges and range shifts of each insect pest species on maize were assessed under the current, the FIO126, the FIO585, the MPI126, and the MPI585 scenarios.

To account for multi-collinearity among the predictors, the following methods were applied. The importance values (IVs) of each variable were calibrated in preliminary species distribution models using the jackknife method (Table S2). A technique recommended by Zhou et al. was adopted to investigate collinearity among predictors for each species individually through a threshold of correlation-coefficient |0.7| (Table S3) [40]. In this method, if predictors obeyed normal distribution, Pearson correlation coefficients were adopted, or Spearman correlation coefficients were used. When collinearity was detected in any pair of variables, the predictor with the lower IV was deleted (Supplementary Material Table S3). Finally, the retained variables were used to build the formal species distribution models and calibrate the ranges for each species.

### 2.4. Building Models

For each species, its ranges were projected using the R package ‘Biomod2’ V.4.1.2, a multi-algorithm platform for species distribution models [39], in which 10 algorithms were initially input (Table S4). The following processes were applied to retrieve pseudo-absences (PAs): if the number of occurrences of a species was ≤1000, 1000 PAs were generated randomly. Otherwise, PAs were randomly generated in numbers equal to the occurrence records [41,42]. Our model reliability was assessed as follows: seven folds of the occurrence records were used to develop the SDMs, while the remaining were reserved for assessing model reliability [41,43]. To ensure model reliability, TSS values (i.e., true skill statistic values) of 0.6 and AUC values (i.e., area under the curve values) of 0.8 were utilized as thresholds for determining whether an algorithm should be included in the Biomod2 ensemble platform (Table S5).

### 2.5. Calibrating Habitat Suitability and Ranges

Spatial maps of habitat suitability for each species under each scenario were initially exported by our models. These maps were then overlapped across scenarios to generate maps of the overlap index of habitat suitability for each scenario. Additionally, all future overlap maps were subtracted from the current ones, resulting in maps of habitat suitability dynamics. Finally, the maximum sum of sensitivity and specificity (MSS) threshold was applied to each species’ habitat suitability maps [44] to determine their ranges. The 24 species’ ranges were then overlapped across scenarios.

### 2.6. Range Dynamic Investigation

Two range dynamic indices were constructed to quantify the range dynamics of each species. The expansion ratio (*ER*) was developed to measure range size alterations between current and future scenarios:

$$ER=\frac{FR}{CR},$$

where *CR* and *FR* represent the current and future ranges, respectively. A higher *ER* indicated greater range expansions in the future relative to current ranges.

Range similarity (*RSI*) was created to reflect alterations in range positions:

$$RSI=\frac{2RS}{CR+FR}$$

where *RS* represents the ranges shared by the future and current ones. A higher *RSI* indicated more similar range positions between current and future scenarios.

Expanding ranges that could potentially be occupied only in the future were also calibrated and subsequently overlapped across scenarios.

## 3. Results

### 3.1. Reliability of the Models

Due to the 10-algorithm platform, our models demonstrated high performance, as evidenced by the high values of AUCs and TSSs. The AUCs varied from 0.922 to 0.976, with an average of 0.959 ± 0.013, while the TSS values varied from 0.715 to 0.861, with an average of 0.812 ± 0.034 (Table S6). These results indicate that our models can reliably project the current and future range shifts in the target species of this present study.

### 3.2. Top Predictors in the Models

**Figure 2:**
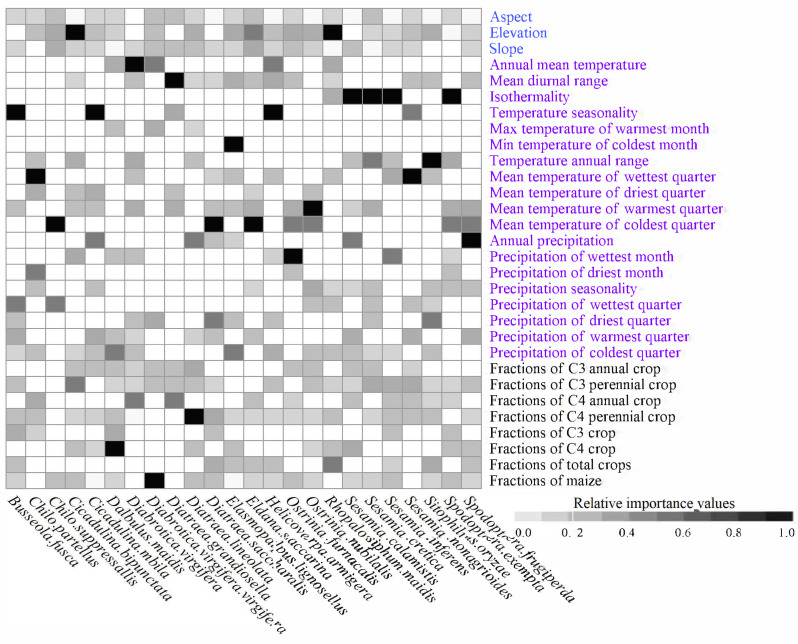
Importance values of each variable in SDMs. We standardized importance values through maximum–minimum method. Relative importance values of each variable were represented by grayscale, and the blanks indicated the variables that were excluded from the formal SDMs.

The top predictors were species-specific (Figure 2 and Table S7). For example, the primary predictors for the range dynamics of *Busseola fusca* (Fuller) (Lepidoptera: Noctuidae) were isothermality (importance value: 0.813), precipitation in the wettest season (0.115), and fractions of cropland (0.026). In contrast, for *D. maidis*, the most important predictors were fractions of C4 crops (0.320), precipitation in the coldest season (0.213), and fractions of C3 crops (0.126), in that order. At the category level, climatic variables were the strongest predictors for the ranges of most species—20 out of 24 species, or 83.3%. In comparison, crop availability predictors had the strongest influence on only three species, i.e., *D. virgifera virgifera*, *D. maidis*, and *Diatraea lineolate* (Walker) (Lepidoptera: Pyralidae), accounting for 12.5% of the total (Supplementary Materials Table S7 and Figure 2). Notably, in the model for *Rhopalosiphum maidis* (Fitch) (Hemiptera: Aphididae), topographical predictors were more influential than climatic and crop availability predictors. (Table S7 and Figure 2).

### 3.3. Dynamics of the Habitat Suitability

For the 24 major maize insect pest species, their spatial patterns of habitat suitability varied greatly, though little variations were detected among those derived by different GCMs for most species (Figure S2). For instance, under most scenarios, we detected high habitat suitability for *Cicadulina mbila* (Naudé) (Homoptera: Cicadellidae) mostly in the southern USA, Mexico, parts of South America (including Venezuela, Ecuador, Peru, Bolivia, Paraguay, Uruguay, and Brazil), sub-Saharan Africa, regions in Asia (such as India, Bangladesh, Myanmar, Thailand, and Southwest China), New Zealand, and Australia, excluding desert areas. However, we identified high habitat suitability of *Chilo suppressalis* (Walker) (Lepidoptera: Crambidae) primarily in Europe, East China, the Korean Peninsula, and Japan. Moreover, their habitat suitability patterns also varied depending on the scenarios (Figure S2). For example, *D. grandiosella* currently showed low habitat suitability in Eastern Europe and Western Russia, while under the F585 scenario, we projected high habitat suitability in these regions (Figure S1).

**Figure 3:**
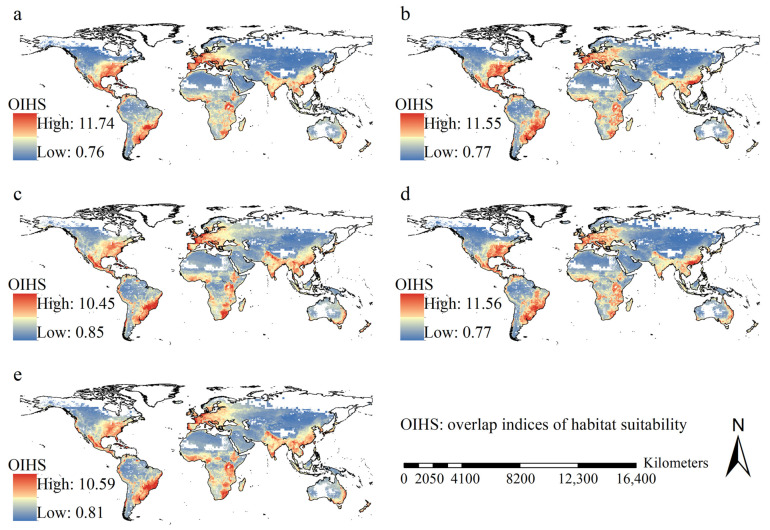
Geographical patterns for the habitat suitability overlap indices. (**a**) current conditions; (**b**) FIO126; (**c**) FIO585; (**d**) MPI126; (**e**) MPI585. High habitat suitability overlap indices were primarily projected in eastern part of the United States of America, Mexico, Brazil, Paraguay, Uruguay, Argentina, and Europe, except for North Europe, southeastern part of Africa, Southeast China, India, the Indochina Peninsula and eastern coast regions of Australia.

**Figure 4:**
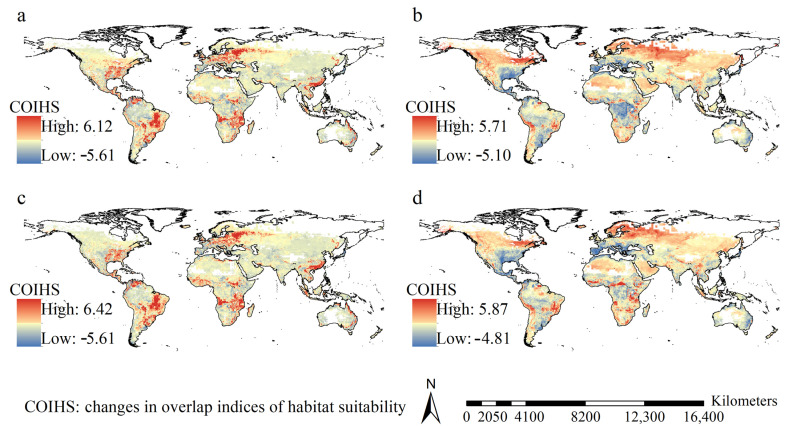
Spatial patterns of the changes in habitat suitability overlap indices. (**a**) FIO126; (**b**) FIO585; (**c**) MPI126; (**d**) MPI585. Under F126 and M126 scenarios. The increases of overlap indices were primarily identified in the eastern regions of USA, Mexico, Venezuela, Brazil, East Europe, West Russia, tropical Africa, eastern regions of China, and eastern coast regions of Australia.

Under most scenarios, high values of habitat suitability overlap indices did not significantly vary with GCMs, and were mostly detected in the east part of the United States, Mexico, Brazil, Paraguay, Uruguay, Argentina, most of Europe except Northern Europe, southeastern Africa, Southeast China, India, the Indochina Peninsula, and coastal regions of East Australia (Figure 3). In other words, under most scenarios, these regions exhibited high habitat suitability. Under the scenarios of FIO126 and MPI126, overlap index increases in habitat suitability were mostly detected in the eastern United States, Mexico, Venezuela, Brazil, Eastern Europe, Western Russia, tropical regions of Africa, East China, and coastal regions of East Australia (Figure 4). However, under the scenarios of FIO585 and MPI585, increased overlap indices were predominantly observed in eastern Canada, Alaska, Northern Europe, and extensive areas of Western Russia (Figure 4).

The regions with increased habitat suitability overlap indices did not significantly vary with GCMs and scenarios, and they covered 95.60, 75.95, 95.65, and 77.58 million km^2^ under the scenarios of FIO126, FIO585, MPI126, and MPI585, respectively. This corresponds to ~70.8%, 56.3%, 70.9%, and 57.5%, respectively, of the global terrestrial area.

### 3.4. Potential Ranges and Their Dynamics

The MSS thresholds that were adopted to estimate potential ranges depended on species (Table S8). For example, the thresholds for *Diabrotica virgifera* (LeConte) (Coleoptera: Chrysomelidae) and *Ostrinia nubilalis* (Hübner) (Lepidoptera: Pyralidae) under the scenario of FIO126 were 0.57 and 0.47, respectively. Additionally, these thresholds were also scenario-specific (Table S8). For example, for *Sesamia nonagrioides* (Lefébvre) (Lepidoptera: Noctuidae) its thresholds under the FIO126, MPI126, and FIO585 scenarios were 0.45, 0.52, and 0.28, respectively. Totally, their MSS thresholds ranged from 0.10 to 0.84, with their average being 0.62 ± 0.13, though they did not significantly vary with GCMs (Table S8).

The potential ranges of insect pests on maize varied by species (Figure S2). For example, for *D. grandiosella*, its potential range under the FIO126 scenario was mostly identified in the United States, covering 1.39 million km^2^ (Figure S2). By contrast, the range for *Sitophilus oryzae* (Linnaeus) (Coleoptera: Curculionidae) under the FIO126 scenario was mostly found in the eastern regions of the United States, Europe, eastern China, Southeast Asia, and southeastern Australia, covering 11.97 million km^2^ (Figure S2). The future ranges of our target species were also scenario-specific (Figure S2). For example, for *D. virgifera*, its potential range under the scenario of FIO126 was mostly identified in Europe (excluding Northern Europe) and the eastern United States, covering 7.11 million km^2^ (Figure S2). However, under the scenario of FIO585, its potential range was primarily found in Europe, such as Northern Europe, as well as the United States and Canada, covering 12.95 million km^2^ (Figure S2).

**Figure 5:**
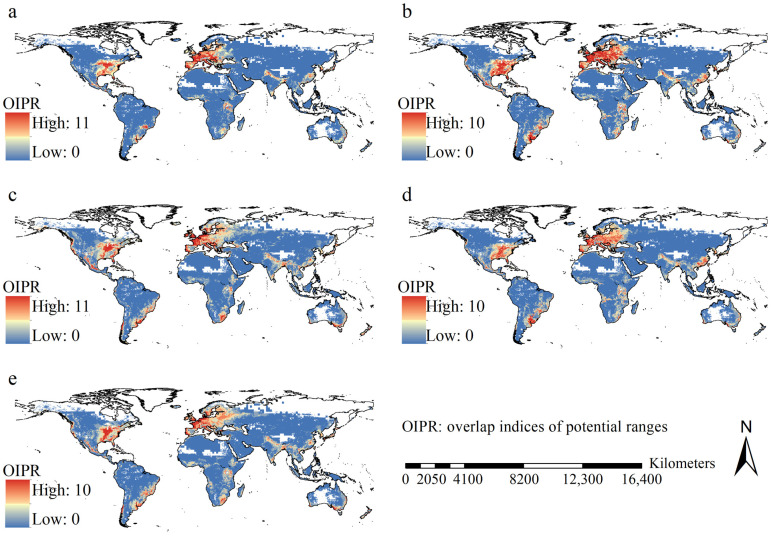
Spatial patterns of overlap indices of potential ranges. (**a**) current scenarios; (**b**) FIO126; (**c**) FIO585; (**d**) MPI126; (**e**) MPI585. High overlapped ranges were mainly projected in eastern regions of USA, Mexico, Europe, the eastern part of China, southeastern regions of South America, Southeast Africa, and the coastline regions of East Australia.

**Figure 6:**
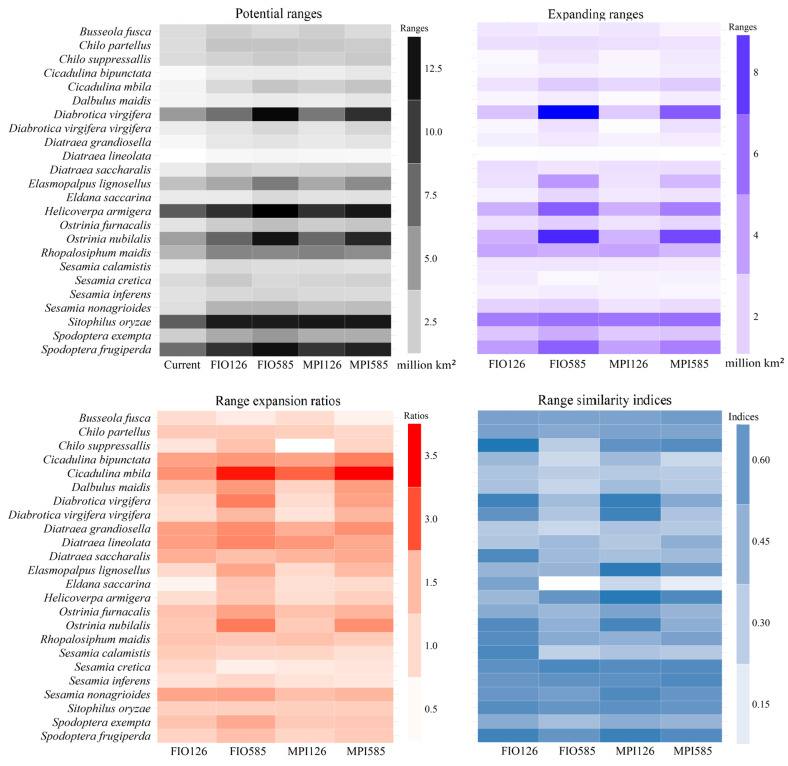
Potential ranges and range shifts in the future. We selected gray, purple, red, and blue to represent the ranges, expanding range, range expanding ratio, and range similarity index, respectively.

Overall, the sizes of the potential ranges for 24 insect pest species on maize varied greatly, ranging from 0.30 to 8.06, 0.64 to 11.97, 0.74 to 13.24, 0.67 to 12.16, and 0.60 to 12.24 million km^2^ under the scenarios of current, FIO126, MPI126, FIO585, and MPI585, respectively. The average range size was 3.96 ± 3.53 million km^2^. Under all five scenarios, higher range overlap indices were projected in the eastern United States, Mexico, Europe, southeastern South America, eastern China, the eastern coastline regions of Australia, and southeastern Africa (Figure 5). Paired-samples *t*-test indicated that their current ranges were significantly smaller than those in the future (all *p* < 0.05). Under all five scenarios, *S. frugiperda*, *Sitophilus oryzae* (Linnaeus) (Coleoptera: Curculionoidea)*,* and *Helicoverpa armigera* (Hübner) (Lepidoptera: Noctuidae) had the largest ranges (Figure 6). Additionally, these observations did not significantly vary with GCMs.

**Figure 7:**
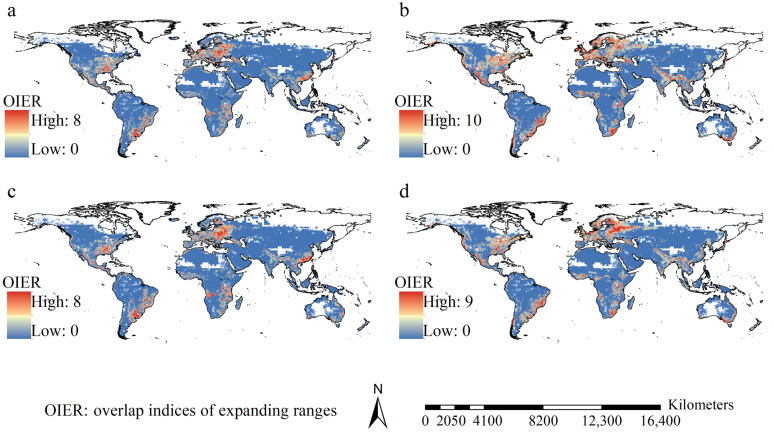
Spatial patterns of overlap indices of expanding ranges. (**a**) FIO126; (**b**) FIO585; (**c**) MPI126; (**d**) MPI585. Overlapped expanding ranges were mostly identified in the eastern regions of USA, Mexico, Europe, Southeast Africa, India, East China, Thailand, Bangladesh, Myanmar, and the eastern coastline regions of Australia.

The expanding ranges varied from 0.48 to 5.51, 0.52 to 9.35, 0.50 to 5.83, and 0.39 to 7.48 million km^2^ under FIO126, MPI126, FIO585, and MPI585, in this order, with an average of 2.33 ± 1.32 million km^2^ (Figure 6). Even though the spatial patterns differed among species, their highest expanding range overlap indices in most future scenarios were projected in Mexico, the eastern United States, Europe, southeastern Africa, India, East China, Thailand, Bangladesh, Myanmar, and the coastline of East Australia (Figure 7). Additionally, *S. oryzae* appeared three times among the top three largest expanding ranges, while other species appeared no more than twice (Figure 6).

The expansion ratios which compared the range sizes between current and future ranges varied across species (Figure 6). These ratios ranged from 1.00 to 2.33, 1.06 to 3.44, 0.84 to 2.89, and 1.01 to 3.50 under the scenarios of FIO126, FIO585, MPI126, and MPI585, respectively, and their average was 1.73 ± 0.49 (Figure 6). Notably, only one of the 96 expansion ratios was smaller than 1.00, and 23 out of 24 species (~95.8%) showed range expansions under all five scenarios (i.e., expansion ratios greater than 1.00). *C. mbila* appeared three times among the top three largest expansion ratios across the five scenarios (Figure 6). The range similarity indices, which measured shifts in range positions, ranged from 0.33 to 0.77, 0.06 to 0.68, 0.26 to 0.76, and 0.15 to 0.67 under the scenarios of FIO126, FIO585, MPI126, and MPI585, respectively, and their average was 0.50 ± 0.15 (Figure 6), suggesting that they did not substantially depend on GCMs. Additionally, 50% of the 96 range similarity indices across all scenarios were smaller than 0.5. Notably, *Eldana saccarina* (Walker) (Lepidoptera: Pyralidae) appeared three times in the list of the five lowest *RSI*s (Figure 6).

## 4. Discussion

The present study retrieved 87,073 global occurrences and developed 24 multi-algorithm models to predict future range shifts in 24 major insect pest species of maize using a unified framework. The results showed that 95.8% of these pest species tended to expand their ranges in the future. Additionally, overlap indices of habitat suitability are expected to increase in most regions. These findings align with previous studies, which also reported range expansions of several insect pests on maize [45,46,47]. Additionally, as far as we know, it might be the first study to investigate the current-future range shift in all 24 major insect pest species on maize within a unified framework, enabling a direct comparison of risks among the species and the identification of the most harmful pests. Furthermore, we analyzed their range and habitat suitability overlaps under future scenarios, providing a comprehensive perspective on the potential impacts of insect pests on global maize production. Additionally, most of these observations did not significantly vary with the two mutually complementary and robust GCMs, partially suggesting the strong robustness of our study. Compared to prior studies, our study provides more valuable and novel knowledge for building strategies to mitigate the impacts of these pests on global maize production in the future.

Besides their range expansions, their range overlaps spatial patterns, indicating that most insect pest species on maize are projected to establish future potential ranges in the eastern United States, Mexico, and other regions. Notably, these areas overlap with major maize-producing regions [23,24,48,49]. Therefore, our study suggests that global maize production will face significant threats from these pests in the future, particularly in these regions, necessitating the development of strict strategies to mitigate their impacts. This conclusion is further supported by various studies that have highlighted the strong potential impact of insect pests on global maize production in the future. However, those studies often based their findings on investigations of one or a few species or employed different methodologies [50,51,52,53,54]. Additionally, as demonstrated by the spatial patterns of their overlap indices, most insect pest species on maize are projected to expand their ranges into the eastern United States, Mexico, Europe, and southeastern Africa, among other regions, under most future scenarios. This suggests that these areas may face higher risks from these pests in the future compared to the present. Additionally, compared with those of single maize pests, the occurrences of this suite of maize pests in these regions would require additional pest management inputs there, which, to a certain extent, was supported by a study by Diffenbaugh et al. [8]. Consequently, stricter strategies than those currently in place should be developed to address the growing threats posed by these insect pest species to maize production in these regions, as their impacts are not yet as pronounced.

Future climate changes strongly influence the range shifts of insect pest species on maize [8,55,56], likely because climatic conditions significantly affect the life history and survival of these pests [57,58,59,60]. However, the exact nature of these effects remains debated. For instance, Senay et al. reported a climate-change-driven range expansion of fall armyworm (*S. frugiperda*) in the future [12]. Conversely, Li et al. projected a decrease in the overall suitable habitats for *Ostrinia furnacalis* (Guenée) (Lep: Pyralidae) and *O. nubilalis*, two major insect pest species on maize [56]. Using a unified scheme, our study demonstrated that, compared with other predictors, climatic variables played stronger roles in the future range expansions of most major maize insect pest species. Therefore, unlike previous studies, our analysis, based on a 10-algorithm platform, indicated that climate-change-driven range expansions in these pest species might represent a general pattern applicable to most maize pests, although further research is required to confirm this. These findings suggest that mitigating future climate changes could be a key strategy for reducing their impact on maize production. Additionally, the limited impact of topography on most species’ potential ranges might be due to several factors. Insect pests on maize are mostly observed in cropland, where topographical patterns could not be mainly characterized by rugged rough terrains, and therefore, the effects of slope and aspects might be very small. The effects of elevation might be reflected by temperature-related predictors, and therefore its influences on the maize pests could be overshadowed.

Our study showed that, compared to host or crop availability, climate predictors could exert stronger effects in the range shifts of 20/24 insect pests on maize, suggesting that they do not closely track their crop hosts. This may arise from their feeding behavior i.e., whether they are monophagous on crop hosts or polyphagous. For instance, *C. bipunctata* feeds not only on maize but also on more than ten poaceous grass species, illustrating its strong polyphagy [33,61]. Another example is the European corn borer (*O. nubilalis*), i.e., in addition to maize, sagebrush (*Artemisia* sp. (Linnaeus)), hop (*Humulus lupulus* (Linnaeus) (Rosales: Cannabaceae)), and mugwort (*Artemisia vulgaris* (Linnaeus) (Asterales: Asteraceae)) also play an important role in its food structure [62]. However, our study also showed that for 3/24 target species, crop availability played stronger roles in their future range shifts than climatic predictors. It may be, to a certain extent, due to their monophagy on crop hosts. For example, *B. maidis* is a maize specialist [63], resulting in its range shifts being more strongly determined by its crop availability than by climatic conditions in our study. Additionally, *D. lineolata* (almost) entirely feed on crop hosts, especially on maize, and no true wild-grass host is known, while its close relative, *Diatraea saccharalis* (Fabricius) (Lepidoptera: Crambidae), has much wider host plants, including a variety of wild grasses [64,65,66]. It might result in one of our observations that the range shifts of *D. lineolata* were more strongly controlled by crop availability than by climatic predictors, while those of *D. saccharalis* were more strongly determined by climatic variables. It, to a certain extent, was supported by a finding by Zhou et al. that the range shifts of *Drosophila suzukii* (Matsumura) (Diptera: Drosophilidae) were more strongly influenced by the availability of its specialist host tree, American Black Cherry (*Prunus serotina*, (Ehrh.) (Rosales: Rosaceae)), in Europe than by climate predictors [40], and another finding by Zhang et al. that the Black Locust availability (*Robinia pseudoacacia* (Linnaeus) Fabales: Fabaceae) showed a greater influence on the range shift in its specialist insect pests in Europe than climate predictors [67]. Overall, the relative influences of climatic predictors and crop availability on their range shifts might be, to a certain extent, determined by whether they are monophagous on crop hosts or not, though we have to acknowledge that further investigation is needed to elucidate this topic in the future

Range size is frequently used in pest risk assessments, as larger potential ranges generally indicate greater impacts. For example, Skendžić et al. argued that the distribution expansion of insect pests could increase the risk of pest-transmitted diseases in crops, biomass losses, and biological control failures, thereby exerting stronger impacts on agricultural production [68]. Our study showed that, compared with other pests, *S. frugiperda* and *H. armigera* were projected to have larger potential ranges under all five scenarios. Based on their range sizes, these three insect pest species pose higher threats to global maize production under all scenarios, highlighting the need for increased attention and management efforts.

In our study, we developed *ER*s to compare the size shifts between future and current ranges, where higher expansion ratios generally indicated greater potential impacts in the future relative to current scenarios. Our analysis revealed that *C. mbila* had the highest *ER*s among most species. Additionally, *S. oryzae* was projected to have a larger expanding range than most species under most scenarios. In other words, these three insect pest species are likely to expand their potential ranges at higher ratios or with greater magnitudes than others. Therefore, it is critical to prioritize the management and control of these three species to mitigate their potential impacts.

In this study, we developed *RSI*s to calibrate their range centroid shifts. Lower indices of range similarity generally indicate more substantial shifts in the range positions where these species could potentially occur. This finding suggests that, to some extent, more significant priority region adjustments need to be incorporated into future strategies to address the impacts of insect pests with low-range similarity indices. Our results revealed that, under all future scenarios, *E. saccarina* exhibited lower range similarity indices compared to most other species. Therefore, to efficiently control its future impacts, we should prioritize more substantial shifts in key regions to combat its effects. Additionally, we noted that under most scenarios, *C. mbila* exhibited the highest *ER*s under most scenarios, suggesting the most significant range expansions. It might be associated with its relatively small potential ranges under the current scenario, which might have implication effects on *ER*s when the current potential range was compared with those under future scenarios. It, to a certain extent, was supported by a finding by Liu et al. [69].

## 5. Conclusions

Here, we built 24 multi-algorithm SDMs to calibrate range shifts in major insect pest species affecting maize and to assess their controlling factors within a unified framework. We observed overall increases in habitat suitability in most regions, along with substantial range expansions there. Both patterns were primarily driven by future climate changes. The relative influence of climate and crop availability on range dynamics could be, to a certain extent, determined by whether they are monophagous on crop hosts or not, though further investigations should be needed on this topic in the future. Future range expansions of major maize pests highlight their potential for increasing impacts on global maize production.

## Data Availability

The original contributions presented in this study are included in the article/Supplementary Material. Further inquiries can be directed to the corresponding authors.

## Supplementary Materials

The following supporting information can be downloaded at https://www.mdpi.com/article/10.3390/insects16060568/s1, Table S1: Major maize insect pest species; Table S2: Importance values of the predictors in preliminary SDMs; Table S3: Multi-collinearity among predictors; Table S4: Ten algorithms for building preliminary SDMs; Table S5: Retained algorithms in formal SDMs; Table S6: Model performance; Table S7: Importance values of all variables in formal SDMs; Table S8: MSS thresholds for determining ranges; Figure S1: Habitat suitability of the 24 major maize insect pest species; Figure S2: Ranges for the 24 target species.
