# Supplementary material for: Future Range Shifts in Major Maize Insect Pests Suggest Their Increasing Impacts on Global Maize Production
Source: Insects. 2025 May 28;16(6):568. doi: 10.3390/insects16060568 (PMC12193563; doi:10.3390/insects16060568)
Supplement: Supplementary file 1 [file insects-16-00568-s001.zip › Figure S1.pdf]

Figure S1 Habitat suitability maps of the 24 target species

Busseola fusca

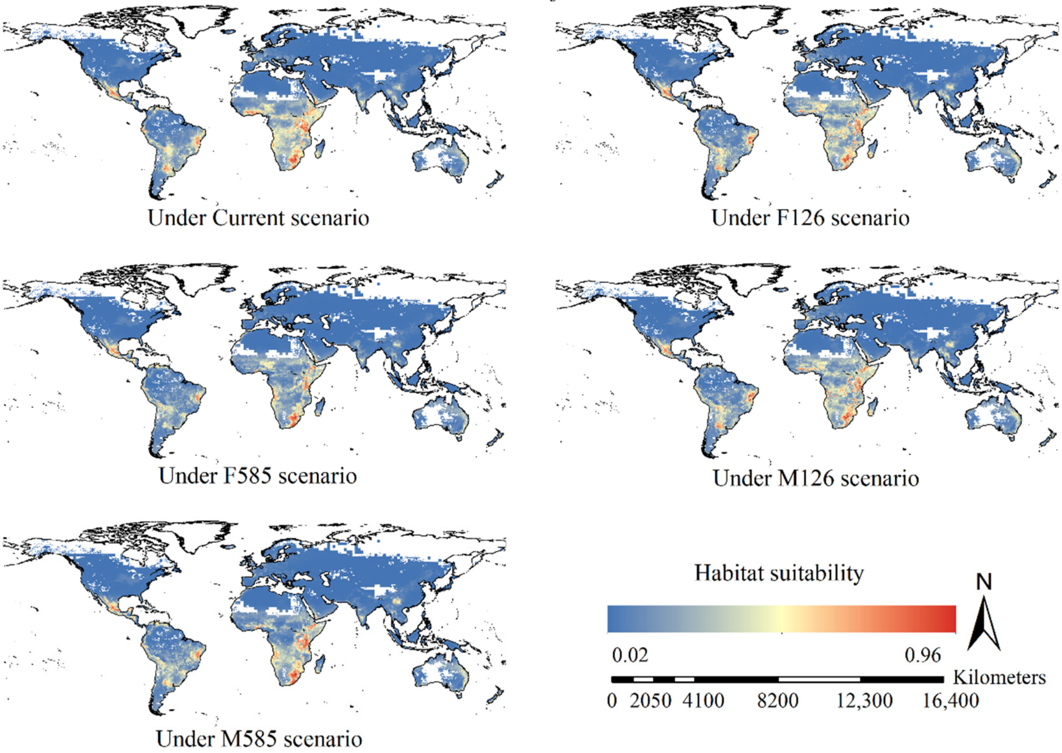

Chilo partellus

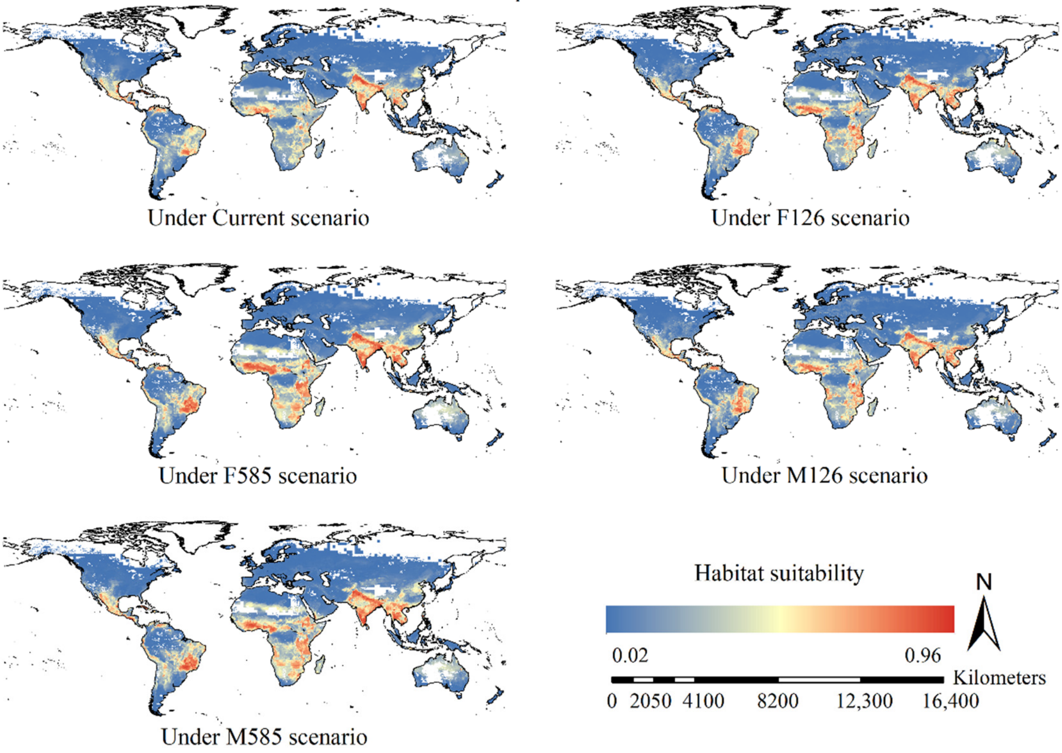

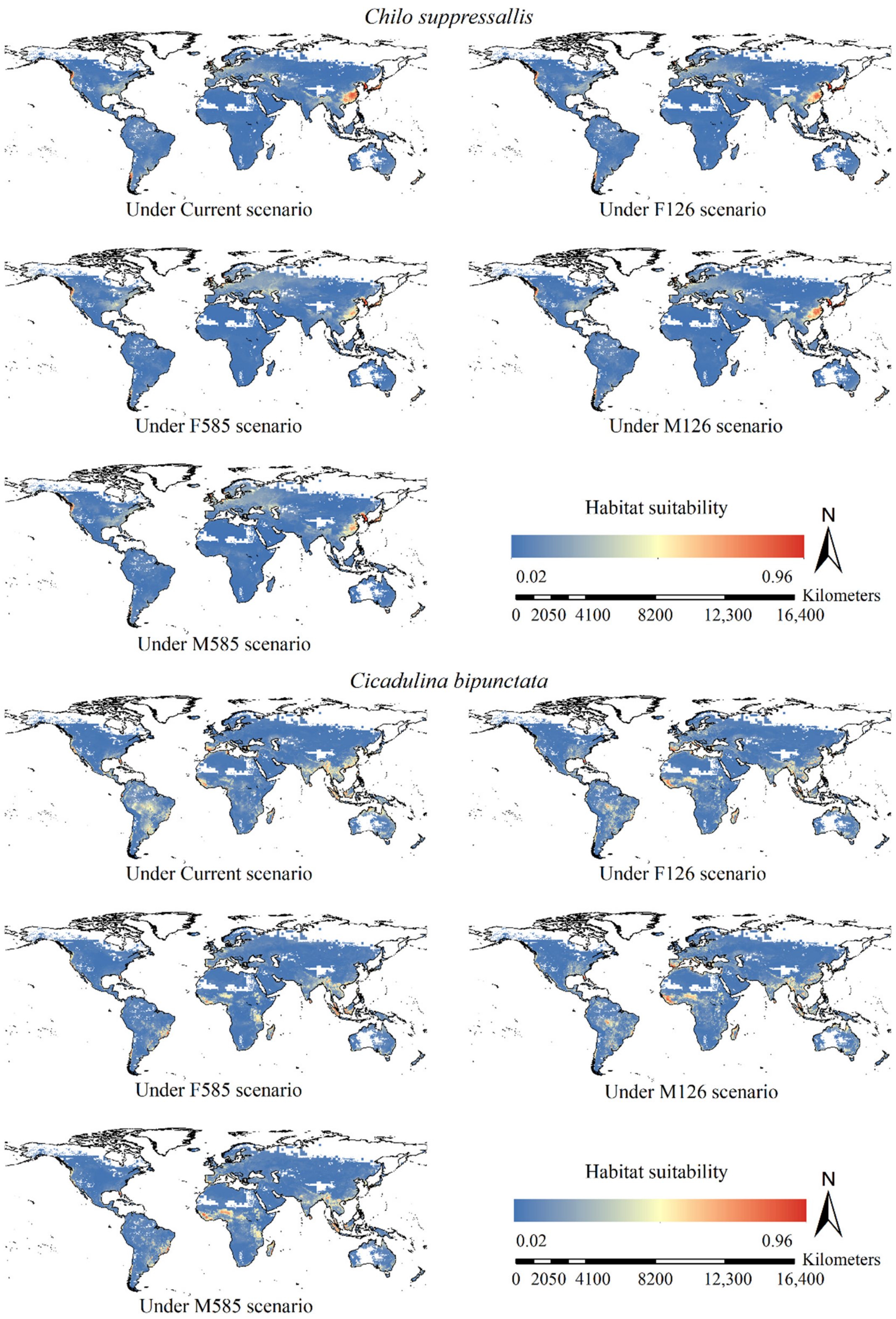
Chilo suppressallis
Under Current scenario
Under F126 scenario
Under F585 scenario
Under M126 scenario
Under M585 scenario
Habitat suitability
0.02
0.96
N
Kilometers
0 2050 4100 8200 12,300 16,400
Cicadulina bipunctata
Under Current scenario
Under F126 scenario
Under F585 scenario
Under M126 scenario
Under M585 scenario
Habitat suitability
0.02
0.96
N
Kilometers
0 2050 4100 8200 12,300 16,400

Cicadulina mbila

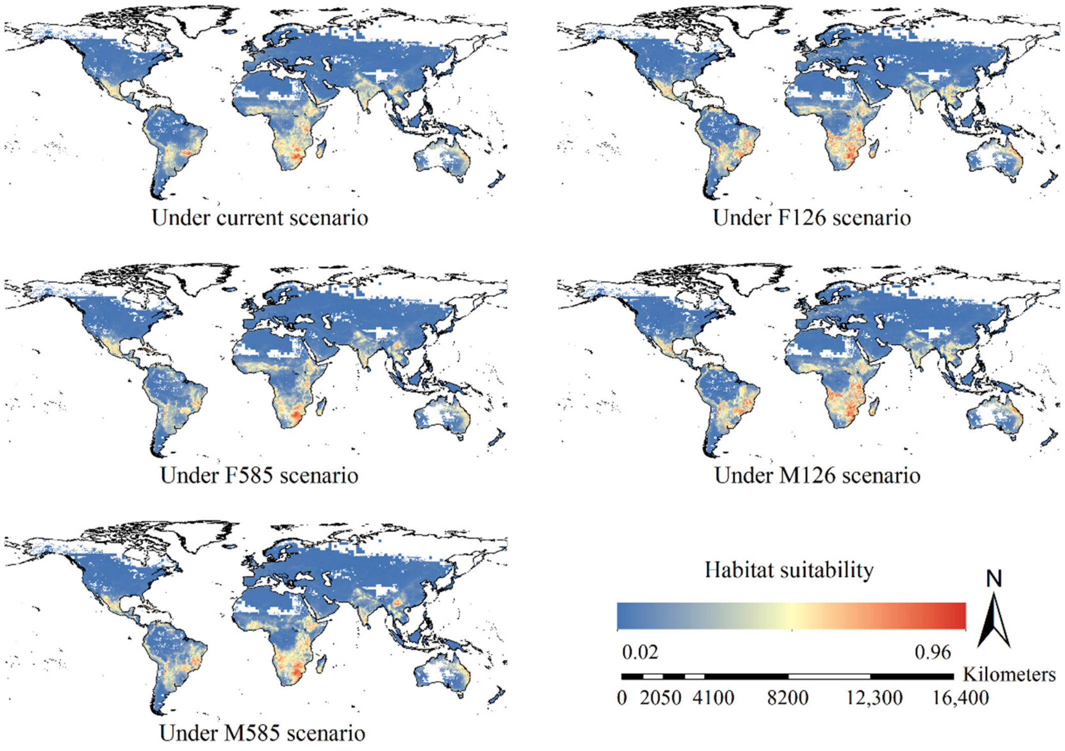

Dalbulus maidis

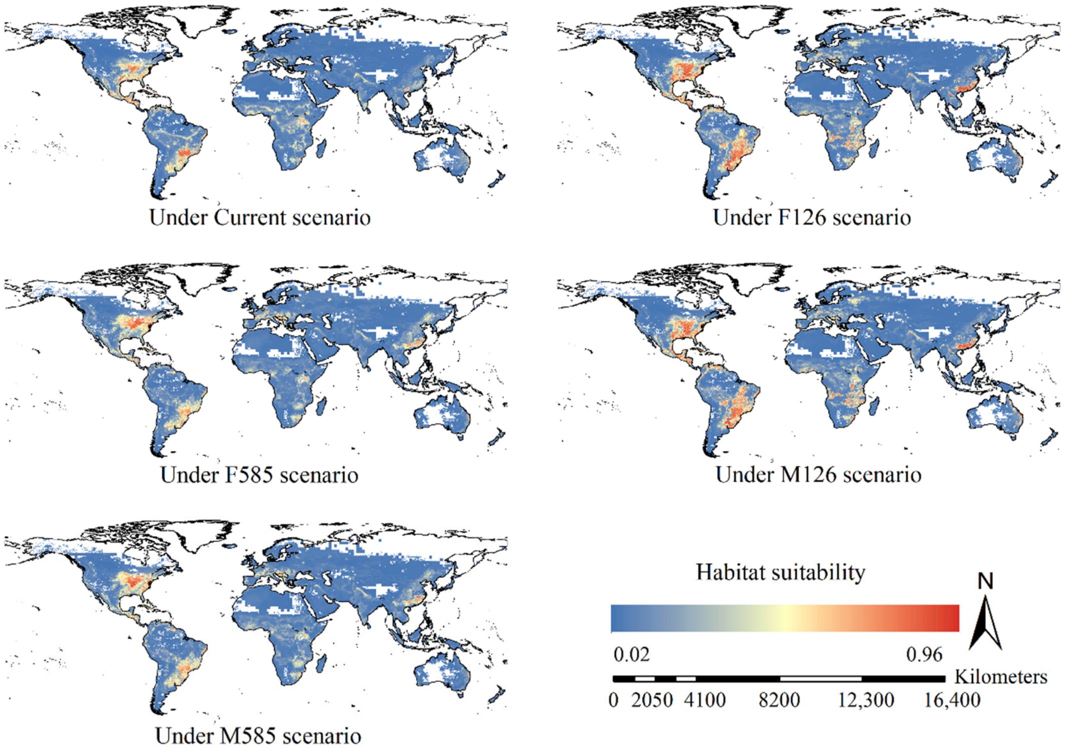

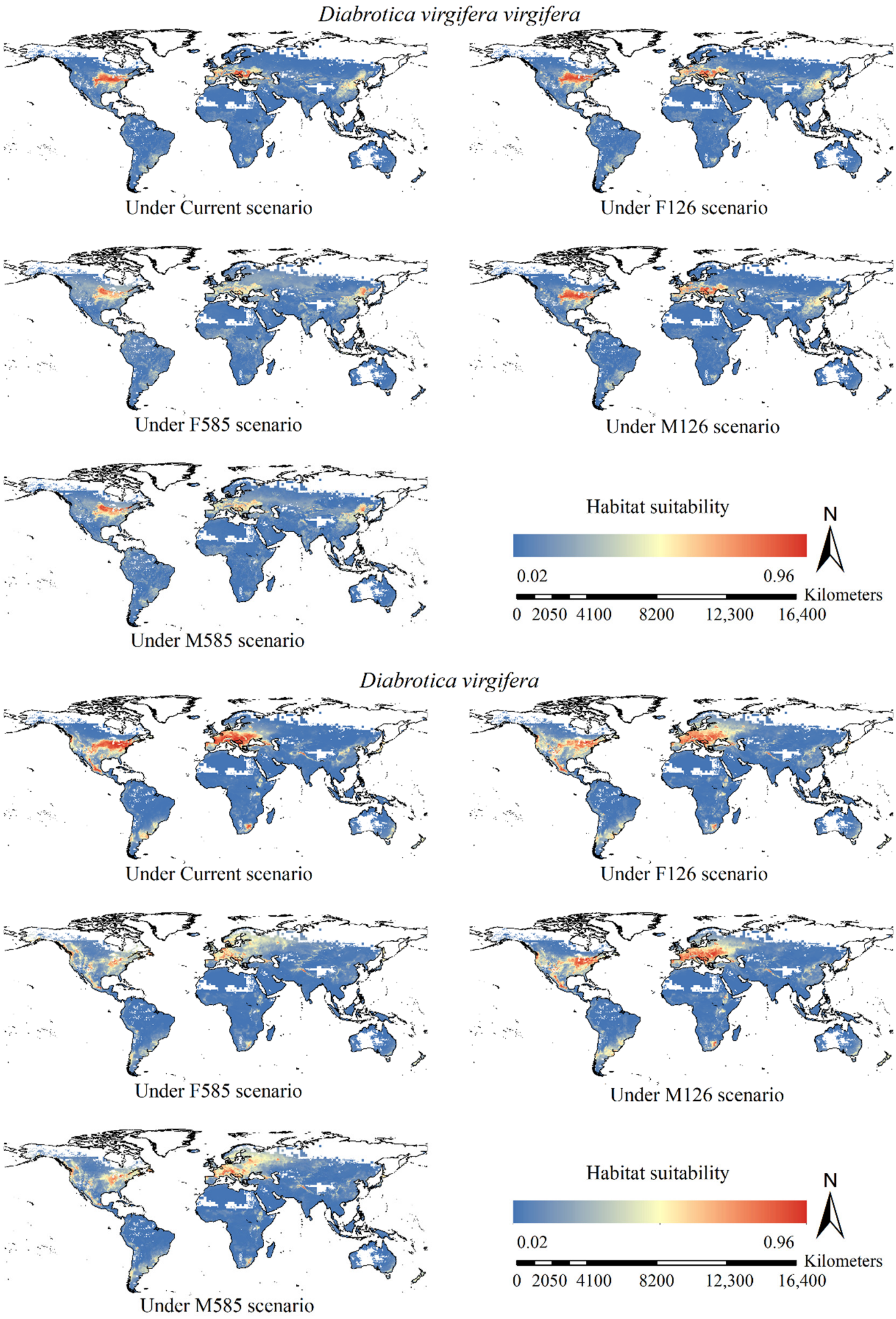
Diabrotica virgifera virgifera
Under Current scenario
Under F126 scenario
Under F585 scenario
Under M126 scenario
Under M585 scenario
Habitat suitability
0.02
0.96
N
Kilometers
0 2050 4100 8200 12,300 16,400
Diabrotica virgifera
Under Current scenario
Under F126 scenario
Under F585 scenario
Under M126 scenario
Under M585 scenario
Habitat suitability
0.02
0.96
N
Kilometers
0 2050 4100 8200 12,300 16,400

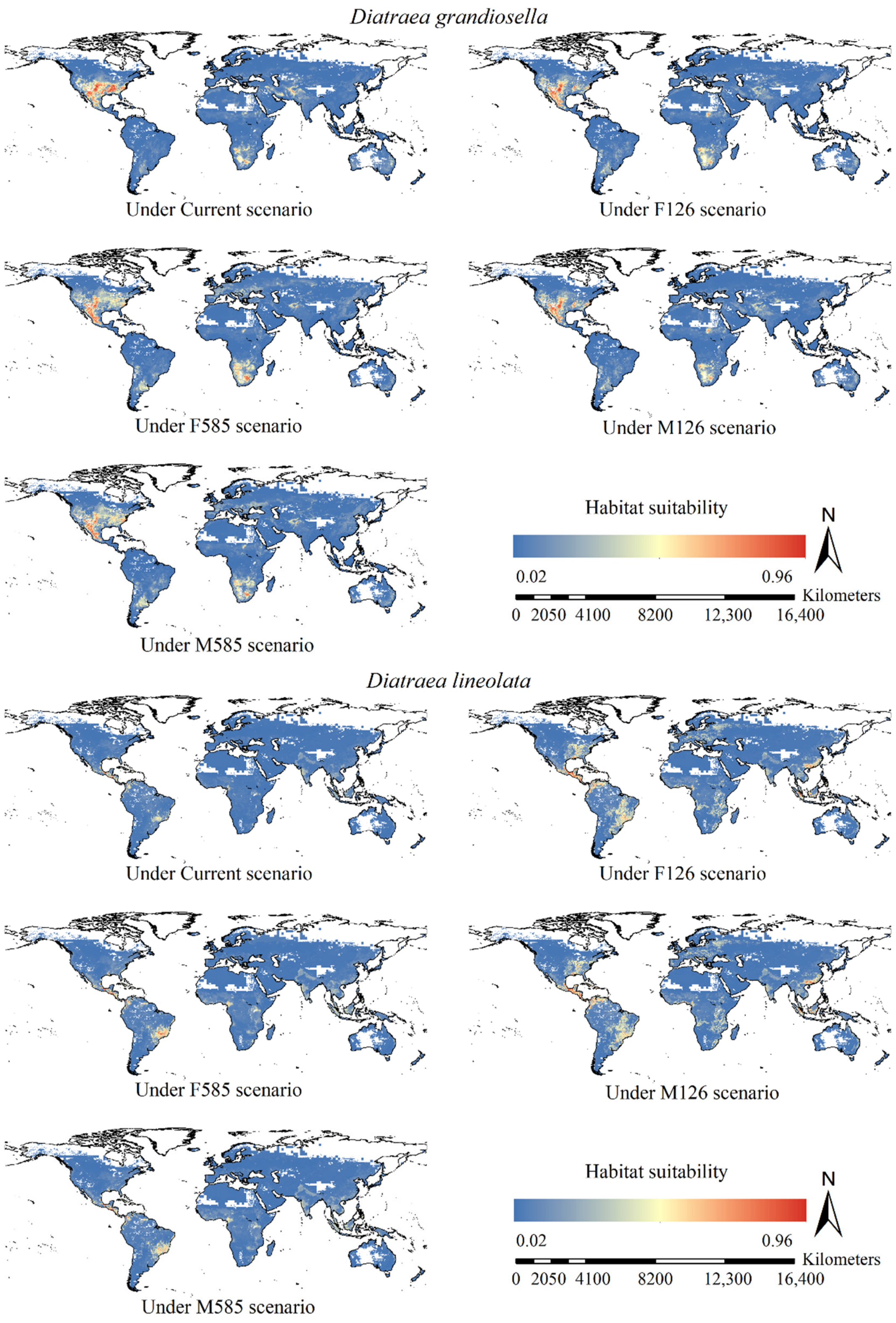
Diatraea grandiosella
Under Current scenario
Under F126 scenario
Under F585 scenario
Under M126 scenario
Under M585 scenario
Habitat suitability
N
0.02
0.96
Kilometers
0 2050 4100 8200 12,300 16,400
Diatraea lineolata
Under Current scenario
Under F126 scenario
Under F585 scenario
Under M126 scenario
Under M585 scenario
Habitat suitability
N
0.02
0.96
Kilometers
0 2050 4100 8200 12,300 16,400

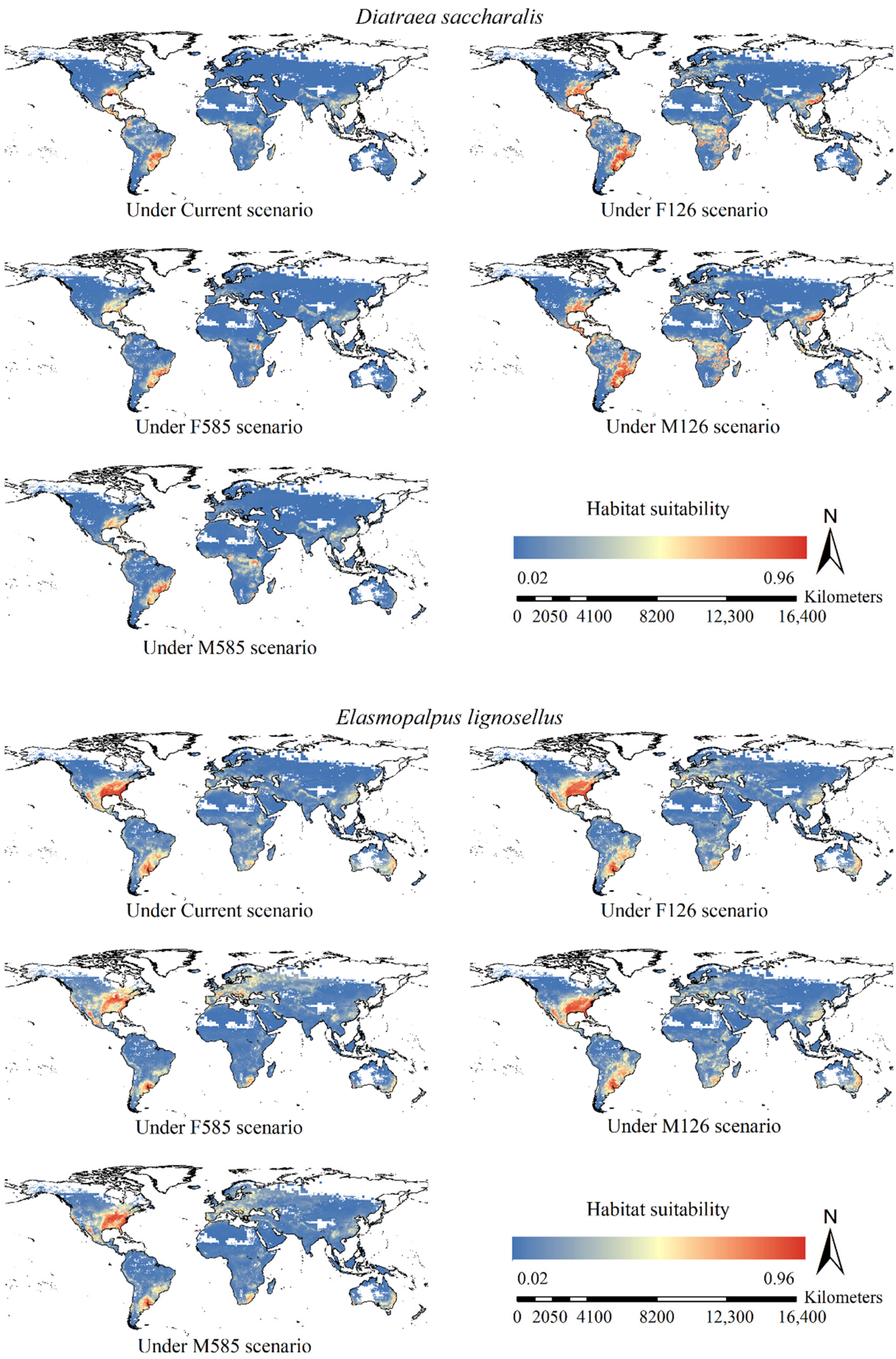

Diatraea saccharalis
Under Current scenario
Under F126 scenario
Under F585 scenario
Under M126 scenario
Under M585 scenario
Habitat suitability
0.02
0.96
N
Kilometers
0 2050 4100 8200 12,300 16,400
Elasmopalpus lignosellus
Under Current scenario
Under F126 scenario
Under F585 scenario
Under M126 scenario
Under M585 scenario
Habitat suitability
0.02
0.96
N
Kilometers
0 2050 4100 8200 12,300 16,400

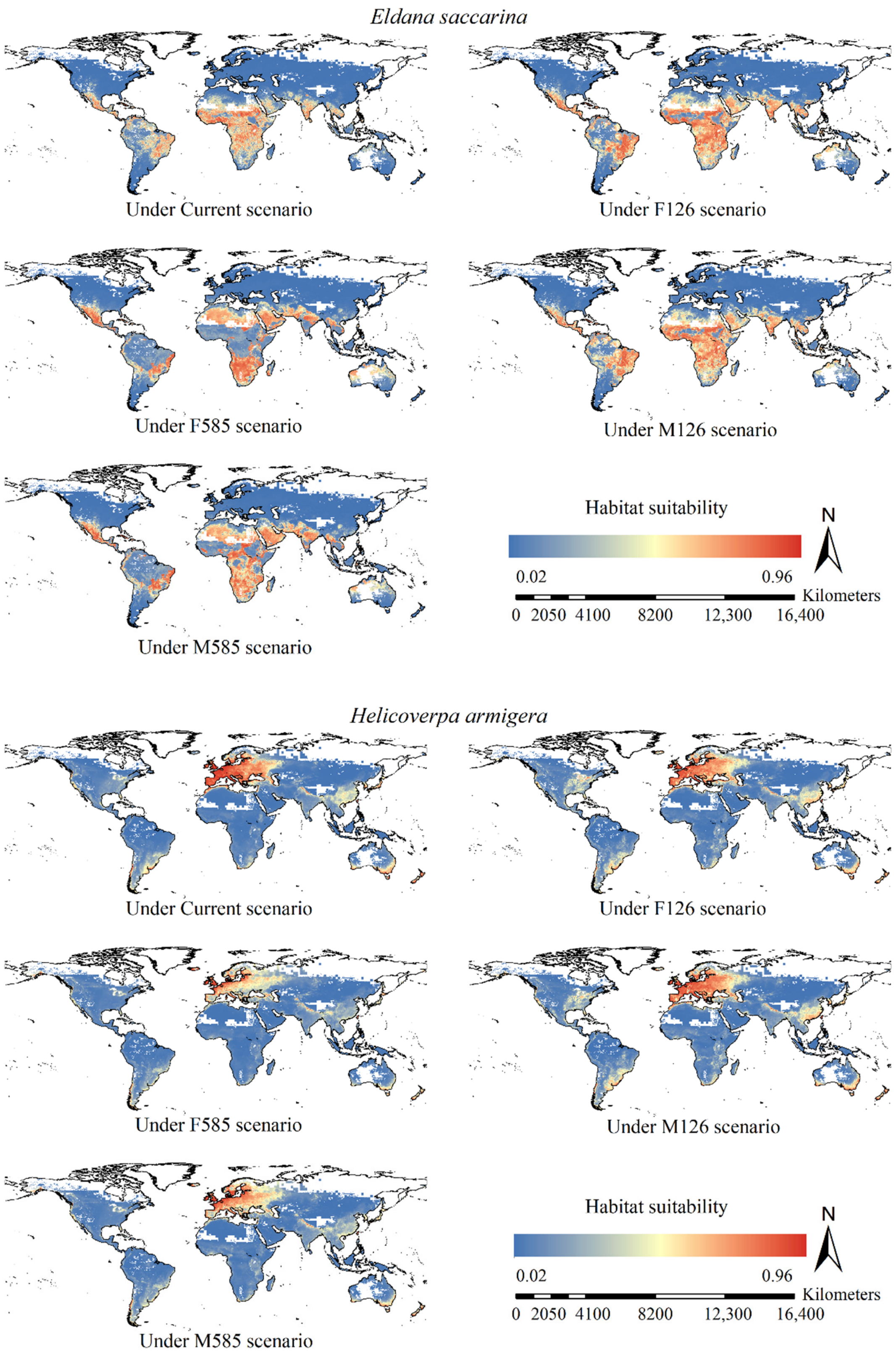
Eldana saccarina
Under Current scenario
Under F126 scenario
Under F585 scenario
Under M126 scenario
Under M585 scenario
Habitat suitability
0.02
0.96
N
Kilometers
0 2050 4100 8200 12,300 16,400
Helicoverpa armigera
Under Current scenario
Under F126 scenario
Under F585 scenario
Under M126 scenario
Under M585 scenario
Habitat suitability
0.02
0.96
N
Kilometers
0 2050 4100 8200 12,300 16,400

Ostrinia furnacalis

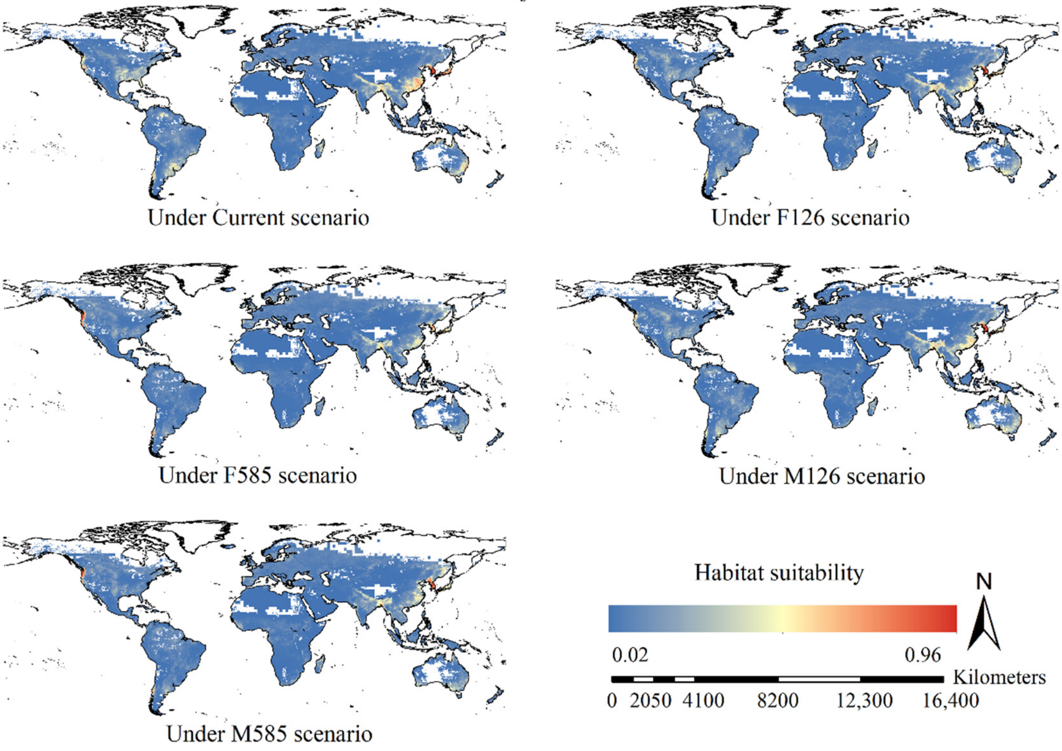

Ostrinia nubilalis

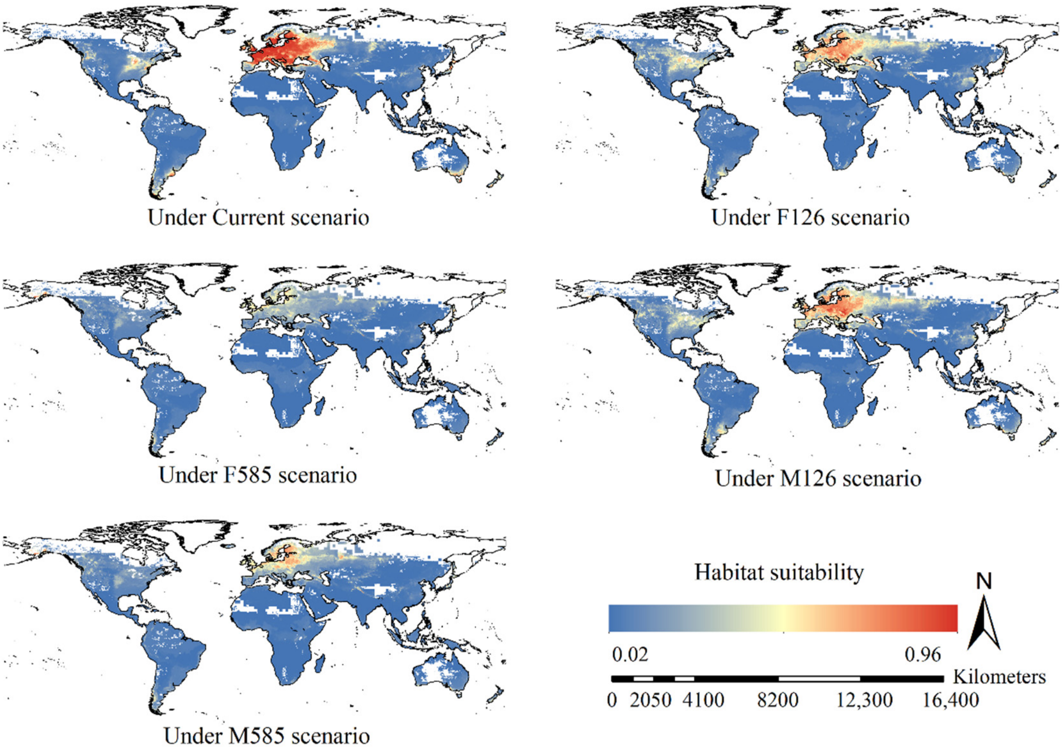

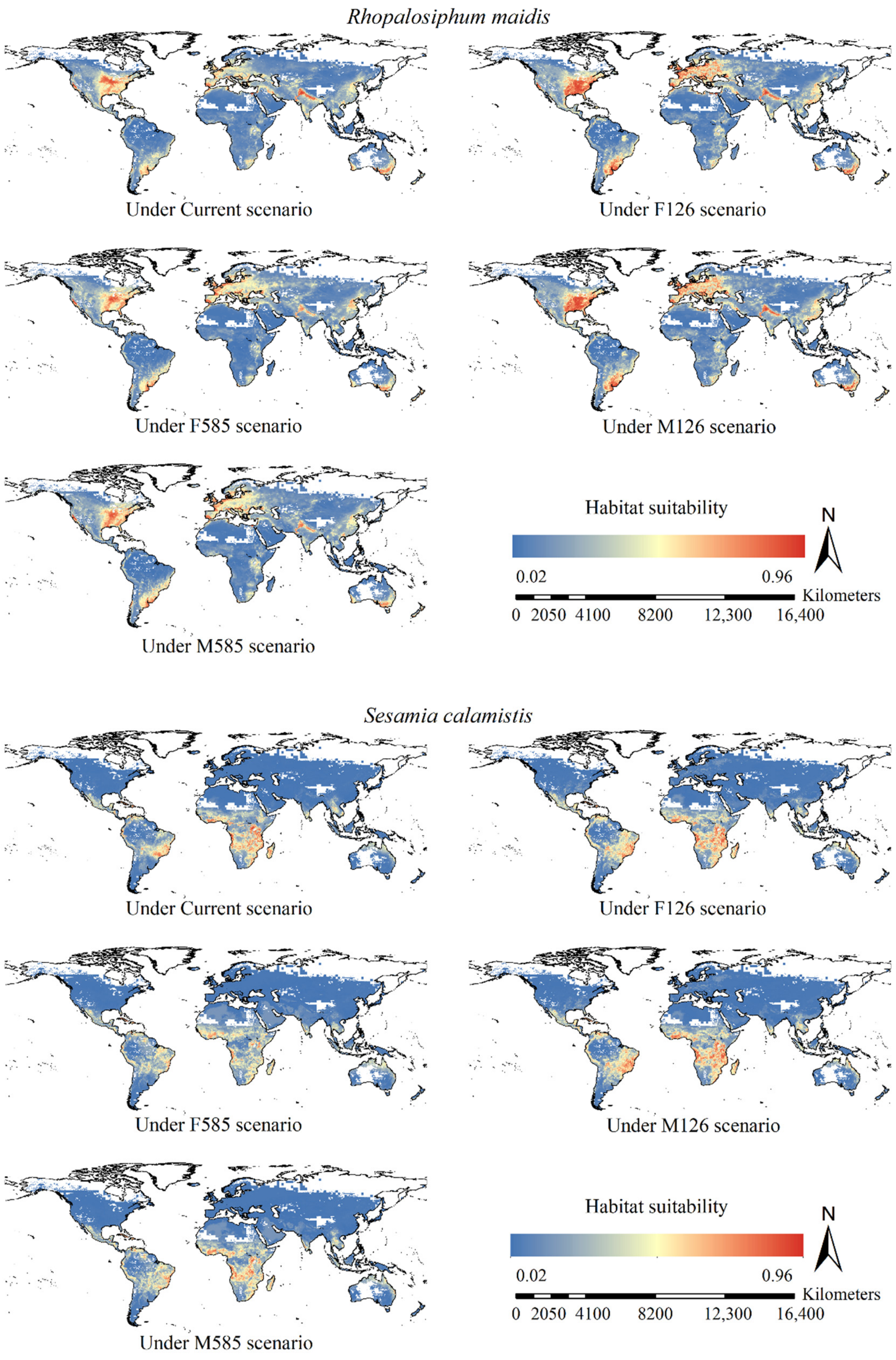

Rhopalosiphum maidis
Under Current scenario
Under F126 scenario
Under F585 scenario
Under M126 scenario
Under M585 scenario
Habitat suitability
0.02
0.96
N
Kilometers
0 2050 4100 8200 12,300 16,400
Sesamia calamistis
Under Current scenario
Under F126 scenario
Under F585 scenario
Under M126 scenario
Under M585 scenario
Habitat suitability
0.02
0.96
N
Kilometers
0 2050 4100 8200 12,300 16,400

Sesamia cretica

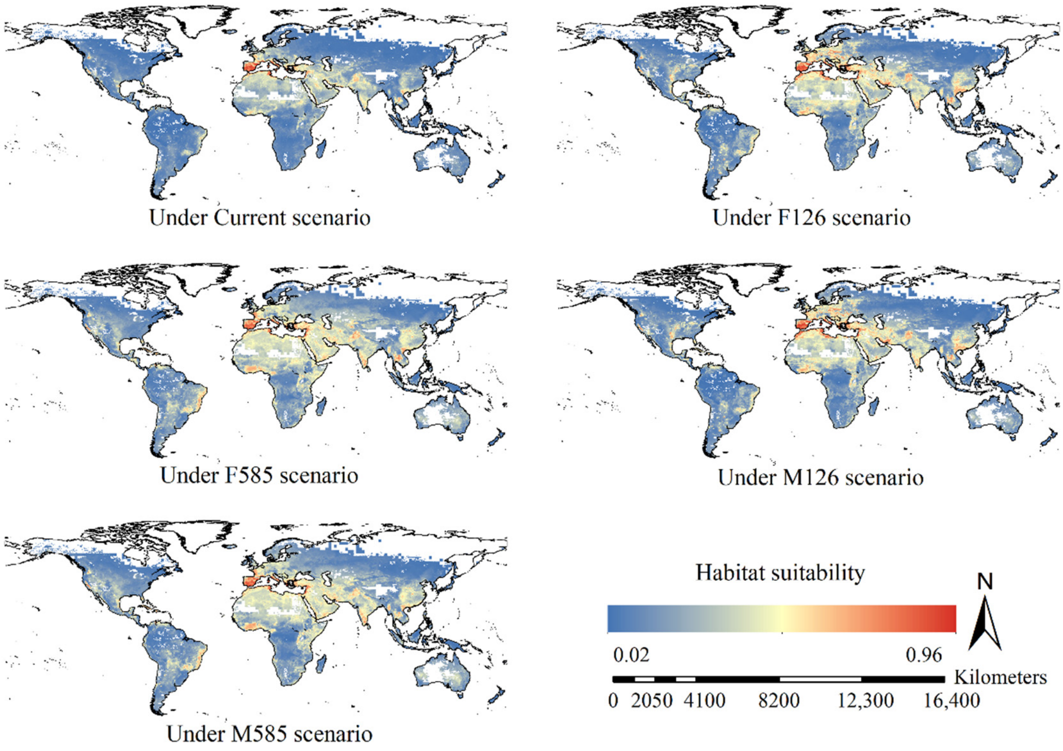

Sesamia inferens

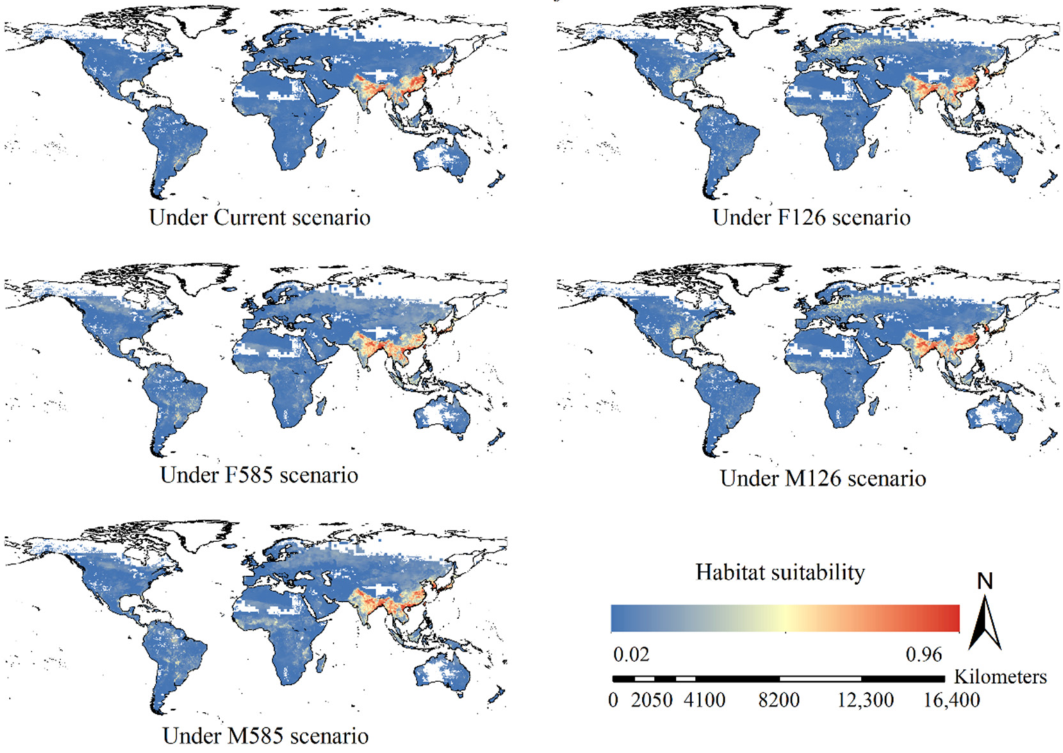

Sesamia nonagrioides

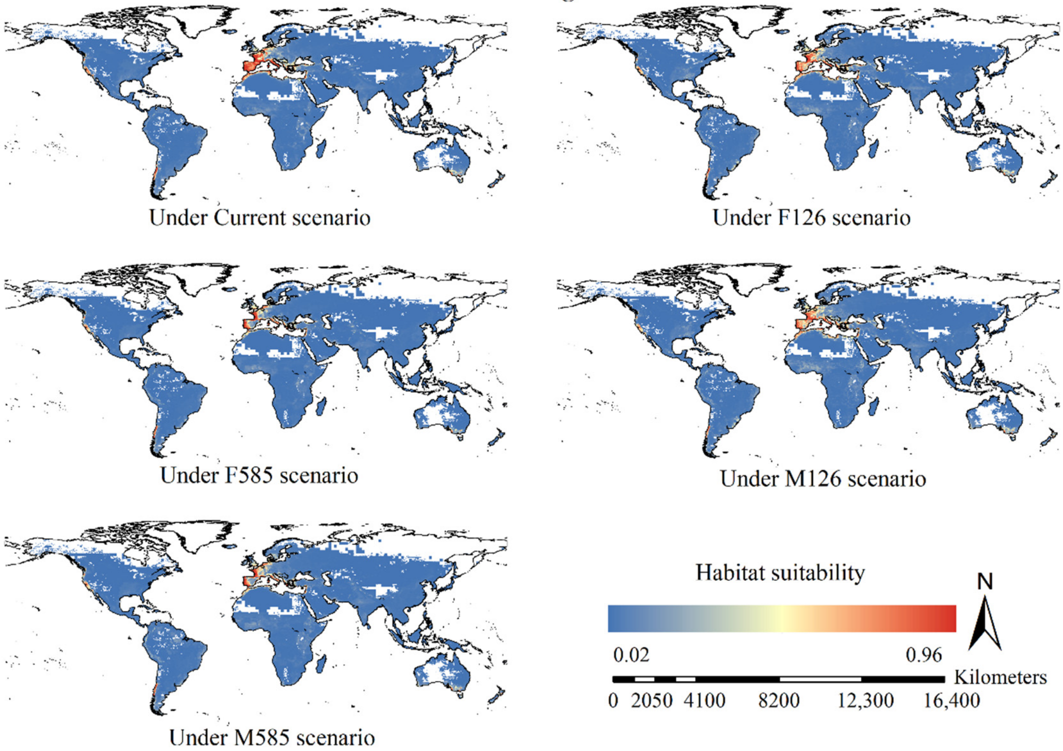

Sitophilus oryzae

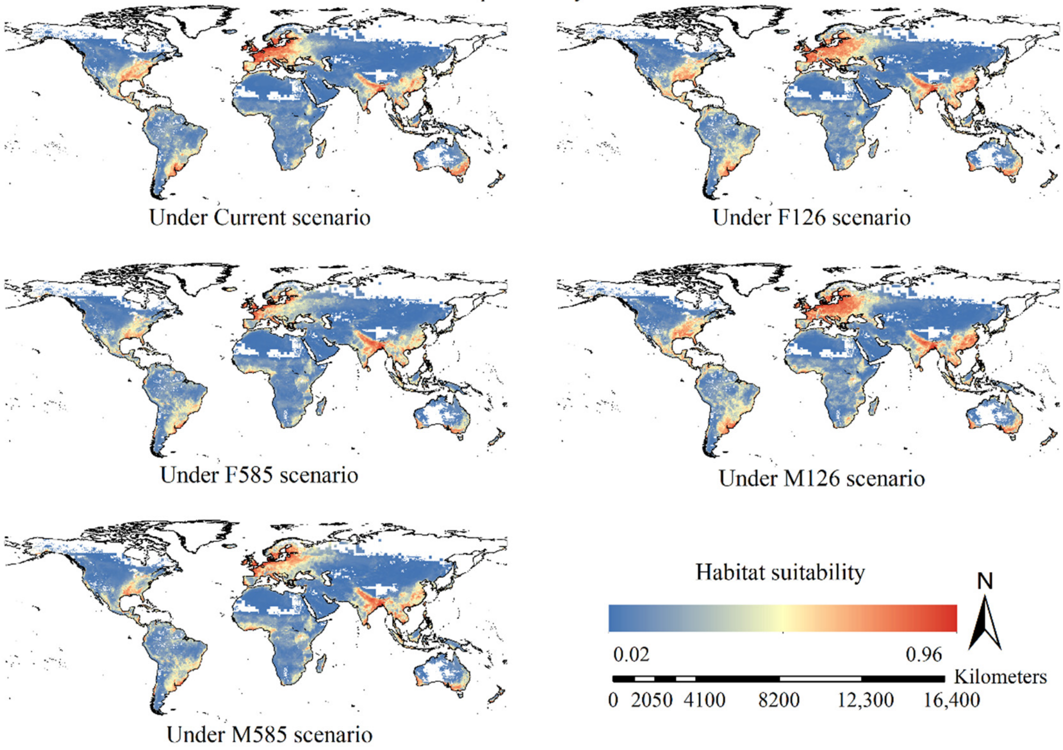

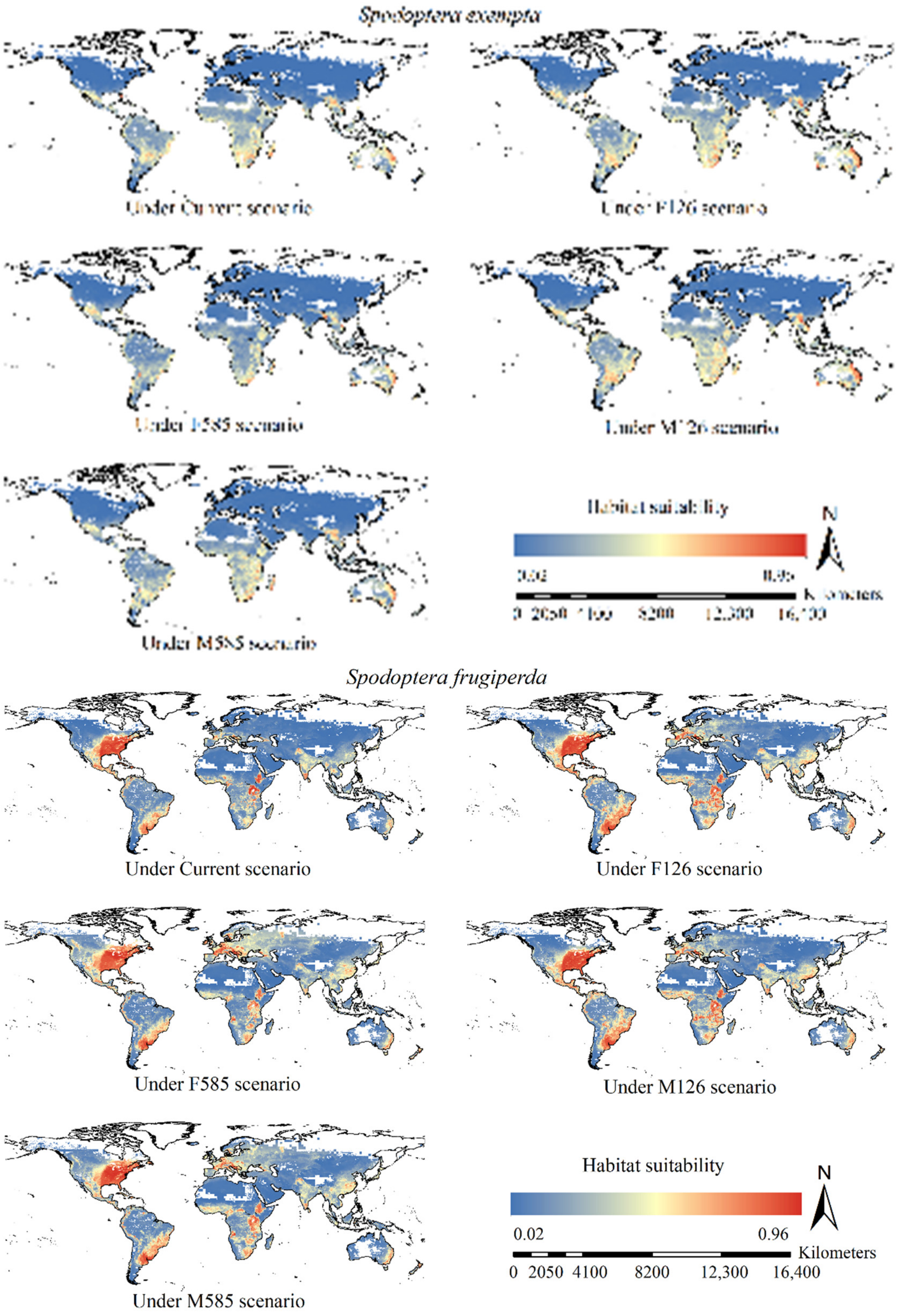
Spodoptera exempta
Spodoptera frugiperda
Under Current scenario
Under F126 scenario
Under F585 scenario
Under M126 scenario
Under M585 scenario
Habitat suitability
0.02
0.96
N
Kilometers
0 2050 4100 8200 12,300 16,400
