# Supplementary material for: Future Range Shifts in Major Maize Insect Pests Suggest Their Increasing Impacts on Global Maize Production
Source: Insects. 2025 May 28;16(6):568. doi: 10.3390/insects16060568 (PMC12193563; doi:10.3390/insects16060568)
Supplement: Supplementary file 1 [file insects-16-00568-s001.zip › Figure S2.pdf]

Figure S2 Ranges for the 24 target species

Busseola fusca

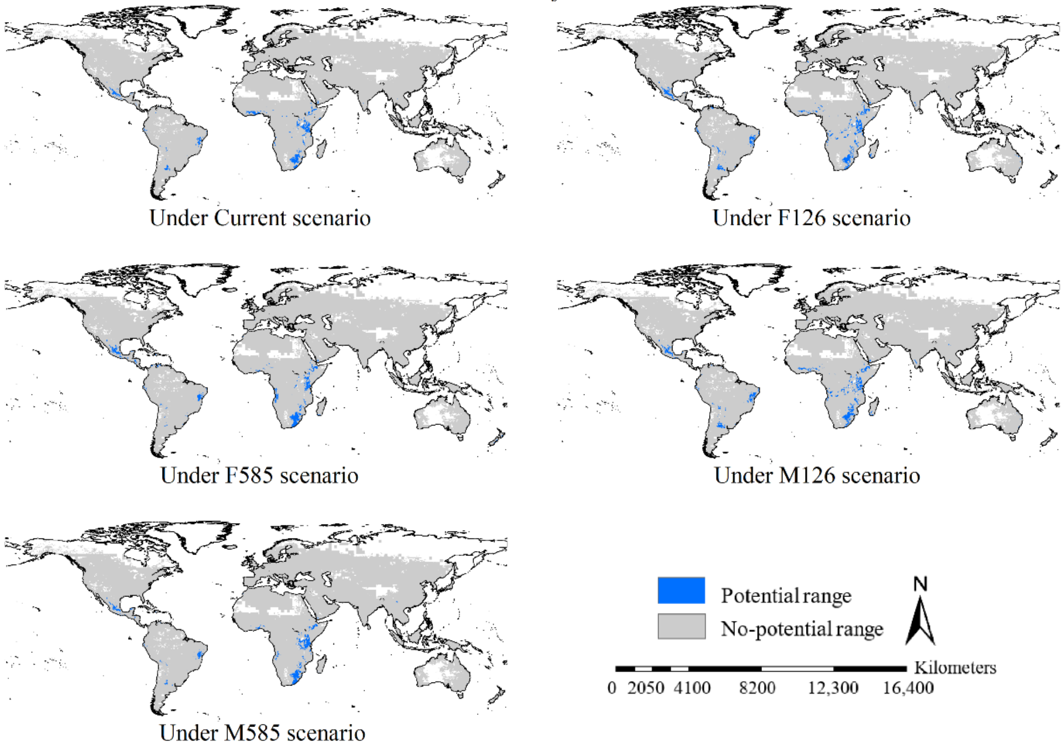

Chilo partellus

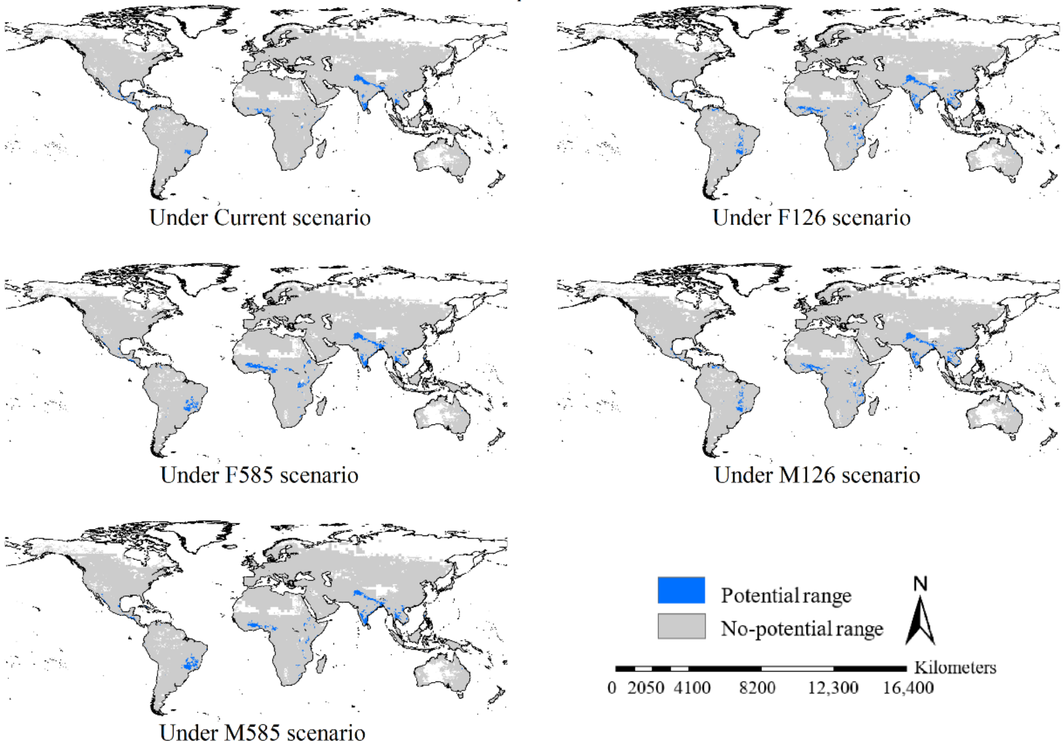

Chilo suppressallis

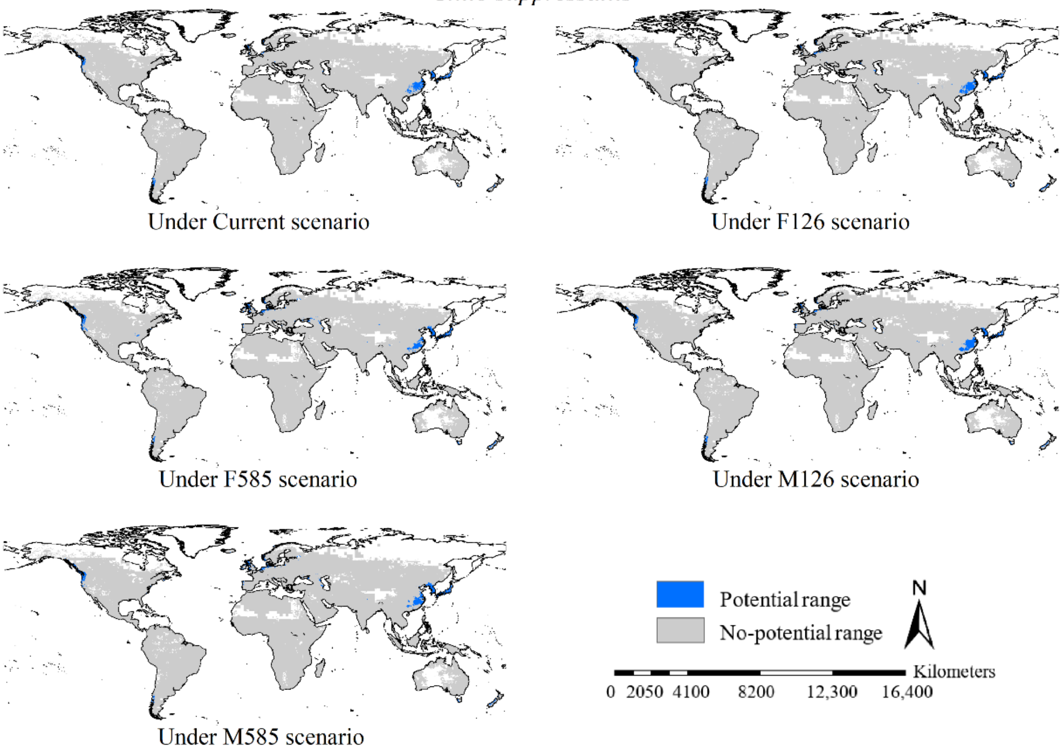

Cicadulina bipunctata

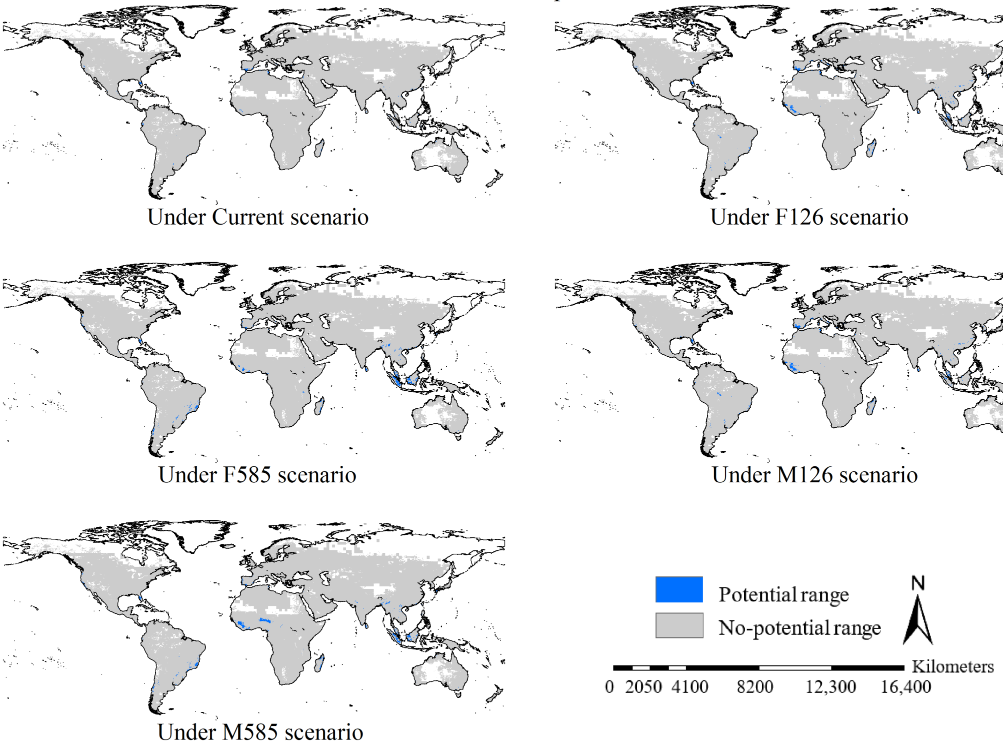

Cicadulina mbila

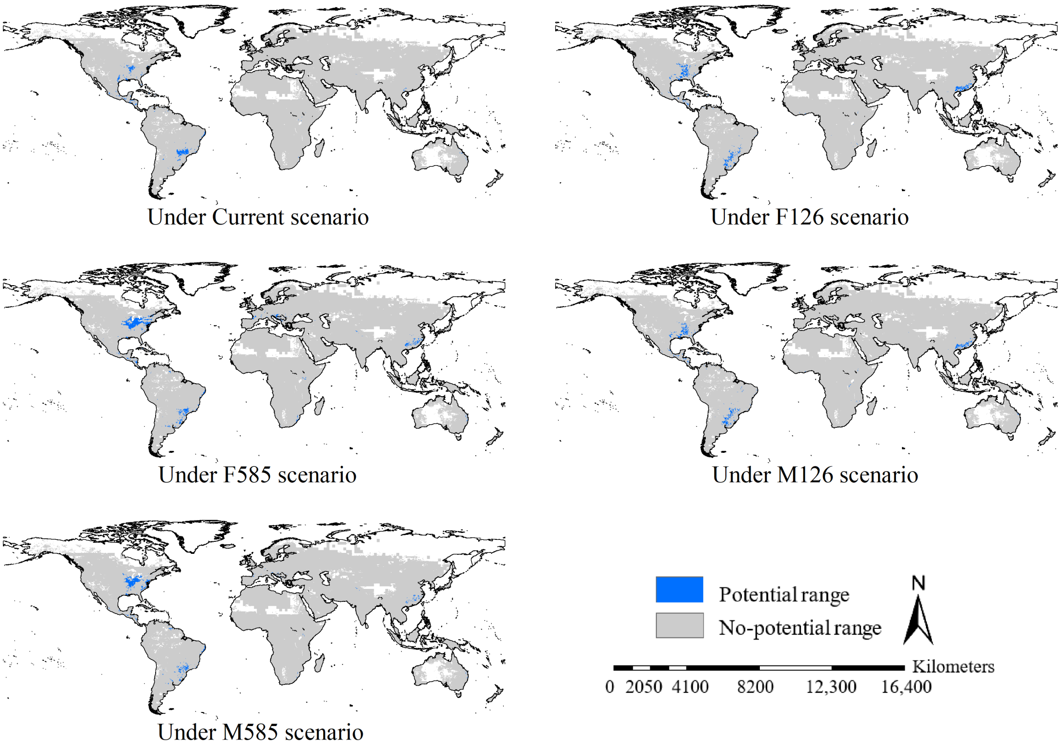

Dalbulus maidis

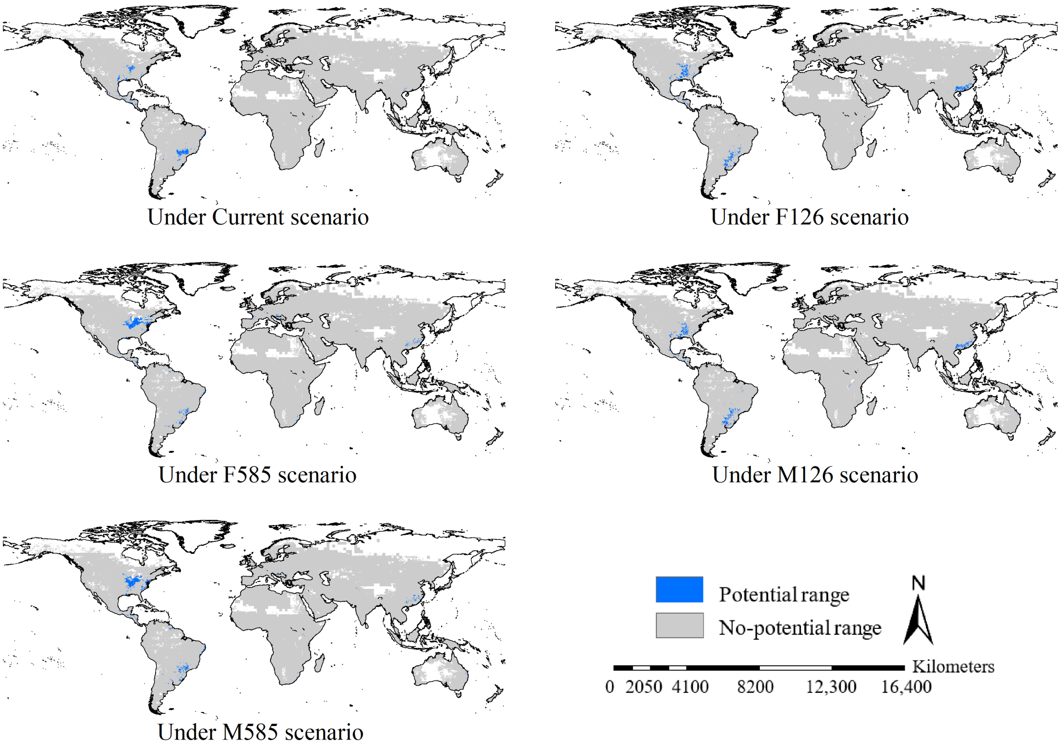

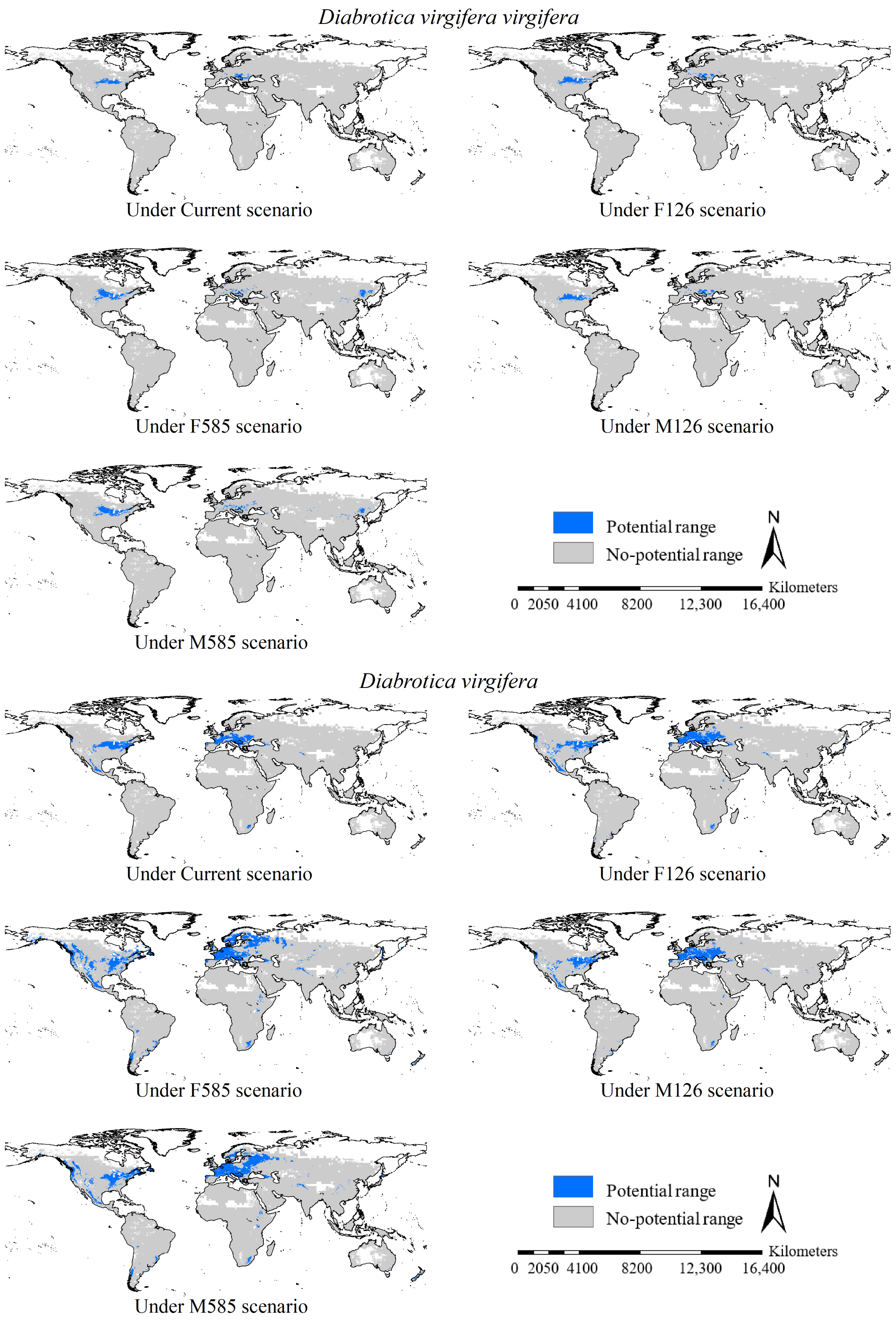
Diabrotica virgifera virgifera
Under Current scenario
Under F126 scenario
Under F585 scenario
Under M126 scenario
Under M585 scenario
Potential range
No-potential range
N
Kilometers
0 2050 4100 8200 12,300 16,400
Diabrotica virgifera
Under Current scenario
Under F126 scenario
Under F585 scenario
Under M126 scenario
Under M585 scenario
Potential range
No-potential range
N
Kilometers
0 2050 4100 8200 12,300 16,400

Diatraea grandiosella

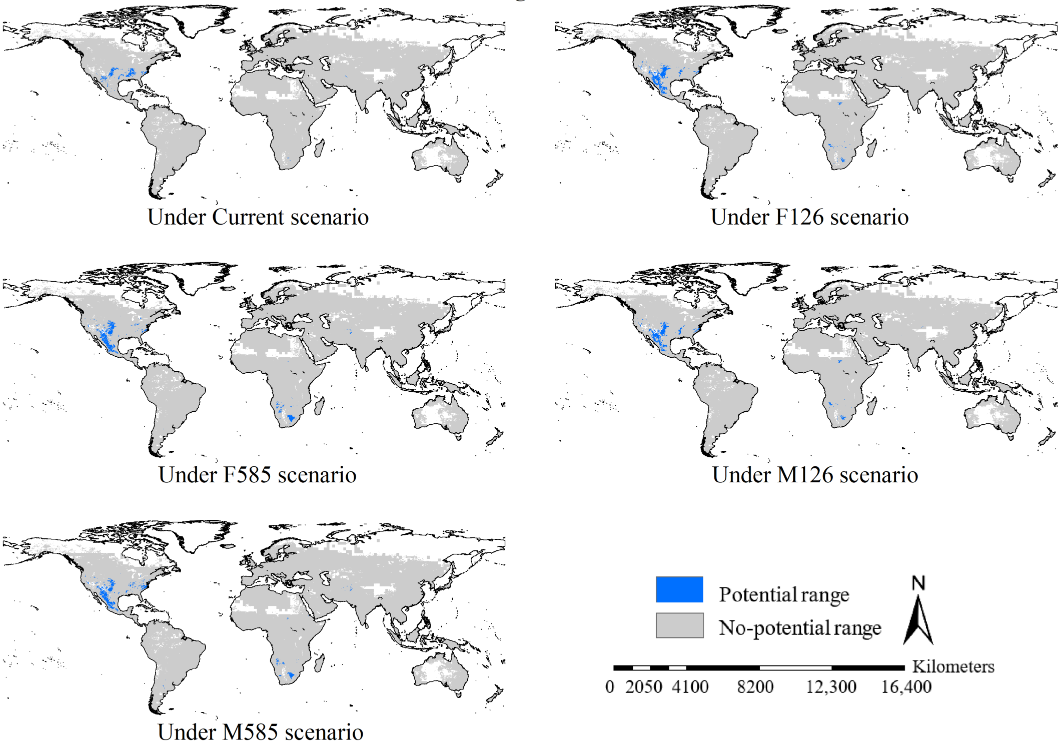

Diatraea lineolata

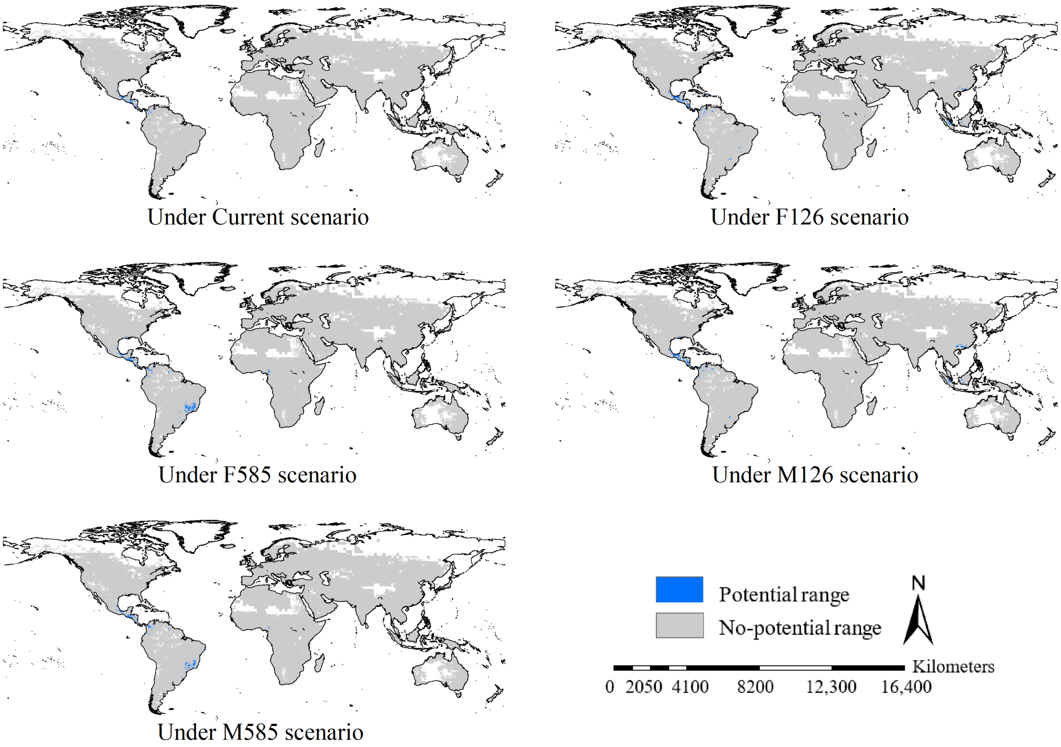

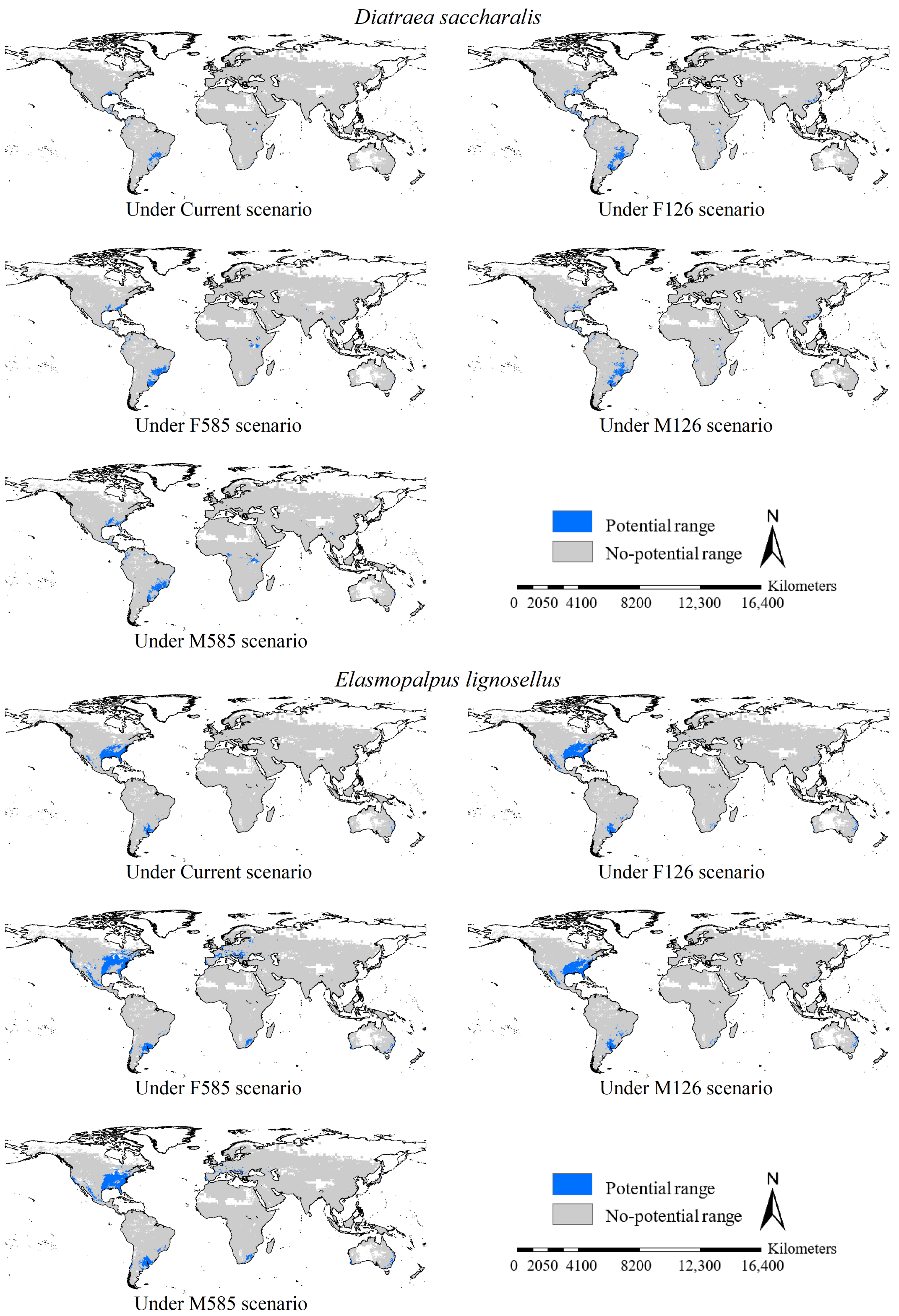

Diatraea saccharalis
Under Current scenario
Under F126 scenario
Under F585 scenario
Under M126 scenario
Under M585 scenario
Potential range
No-potential range
N
Kilometers
0 2050 4100 8200 12,300 16,400
Elasmopalpus lignosellus
Under Current scenario
Under F126 scenario
Under F585 scenario
Under M126 scenario
Under M585 scenario
Potential range
No-potential range
N
Kilometers
0 2050 4100 8200 12,300 16,400

Eldana saccarina

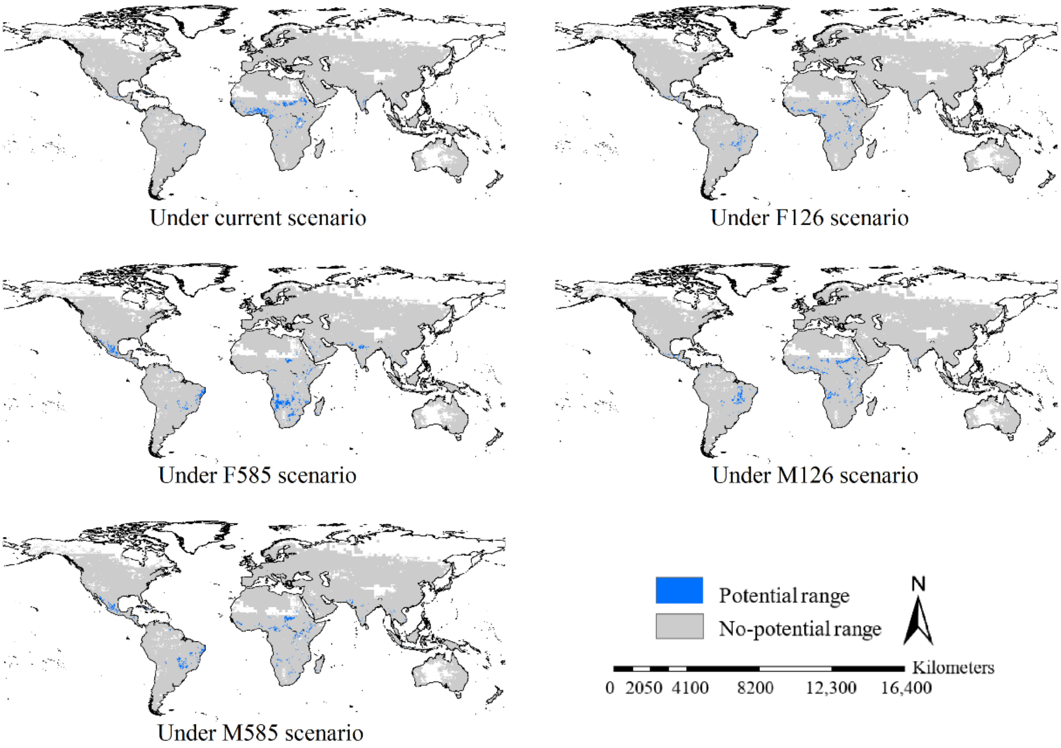

Helicoverpa armigera

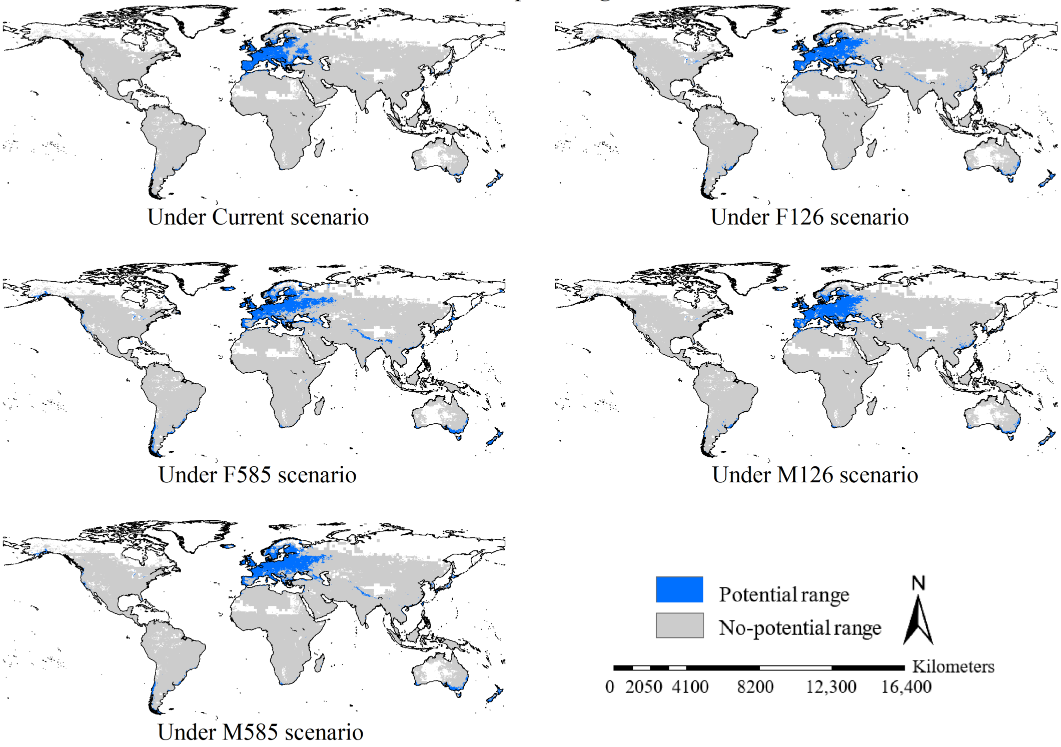

Ostrinia furnacalis

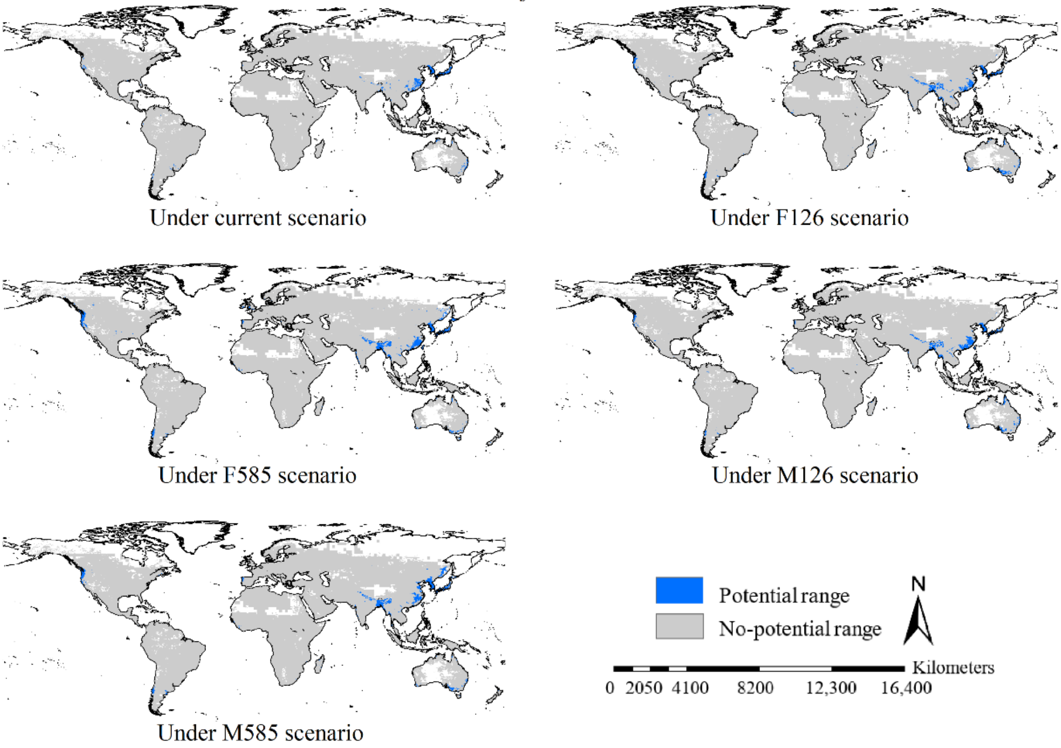

Ostrinia nubilalis

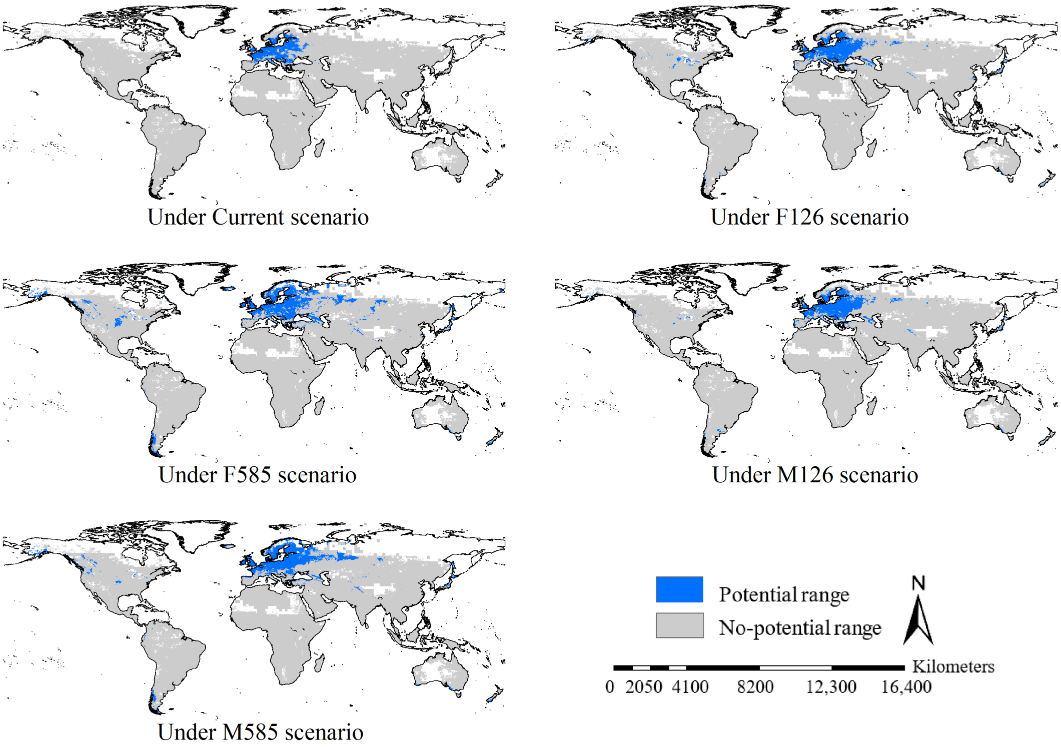

Rhopalosiphum maidis

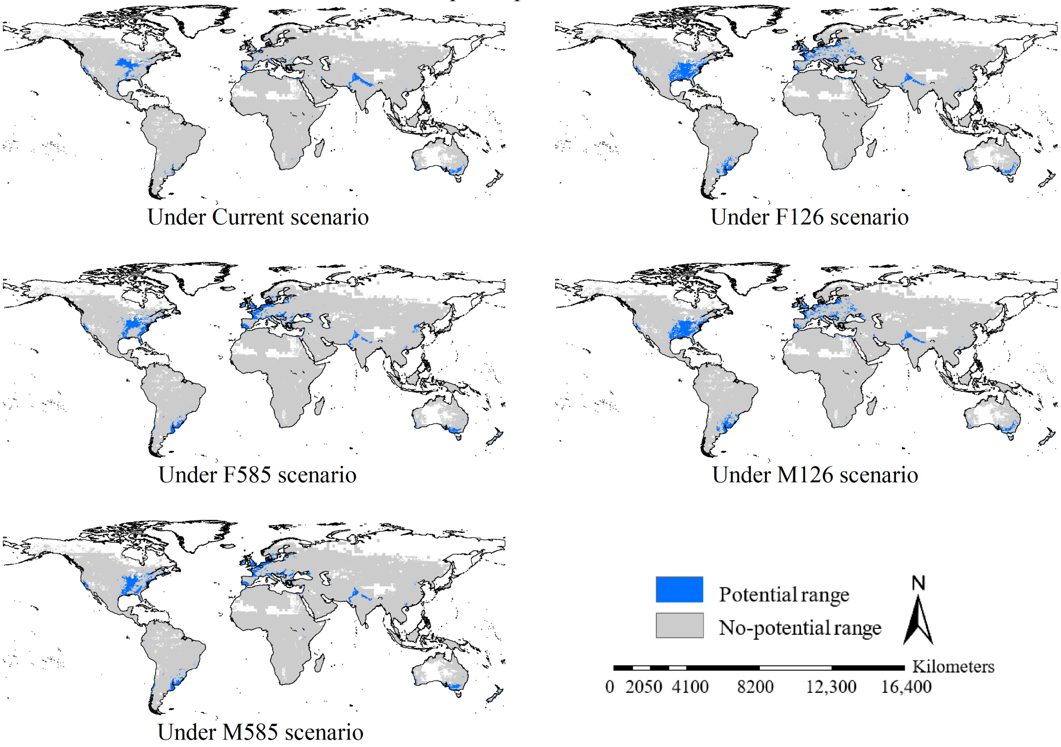

Sesamia calamistis

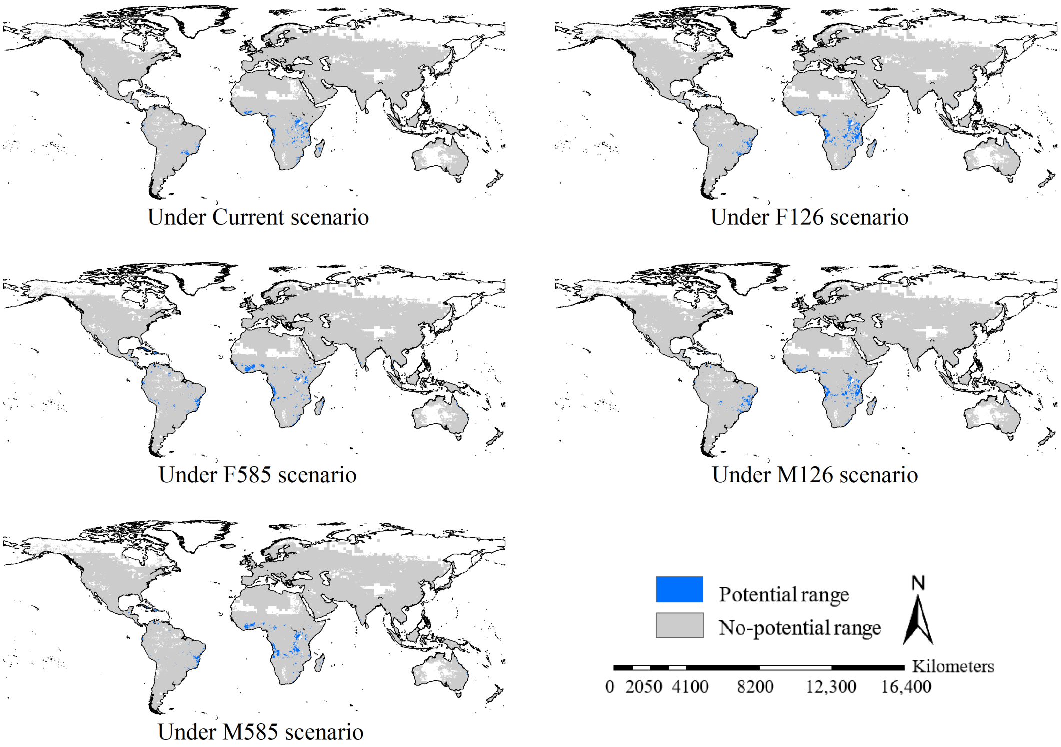

Sesamia cretica

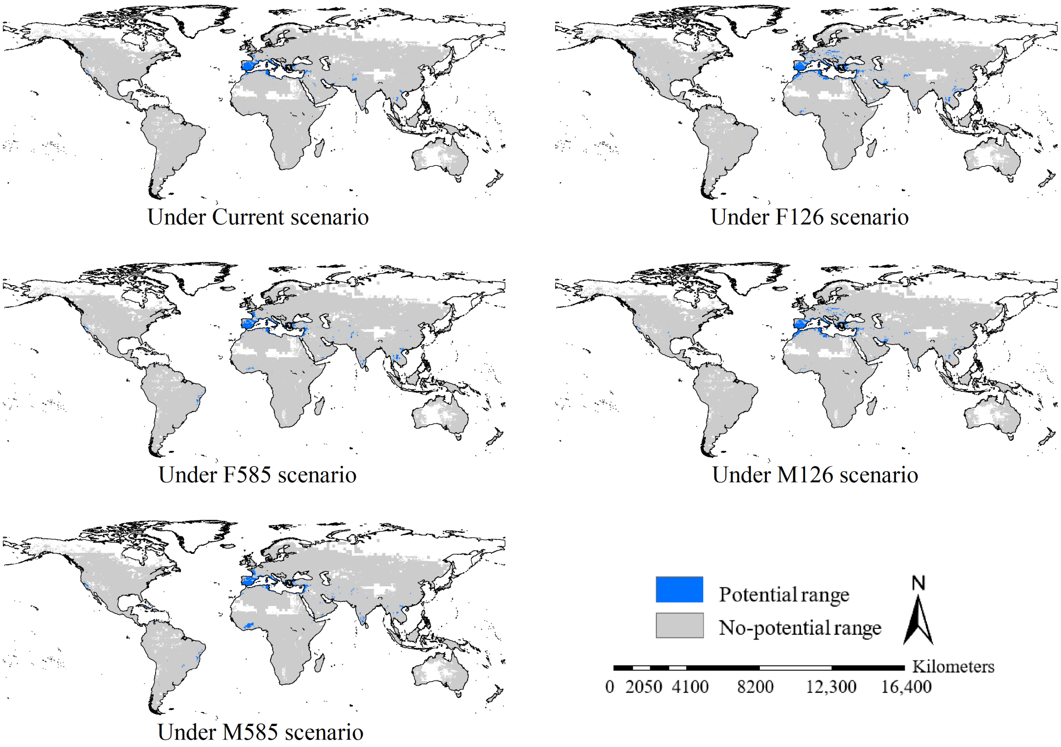

Sesamia inferens

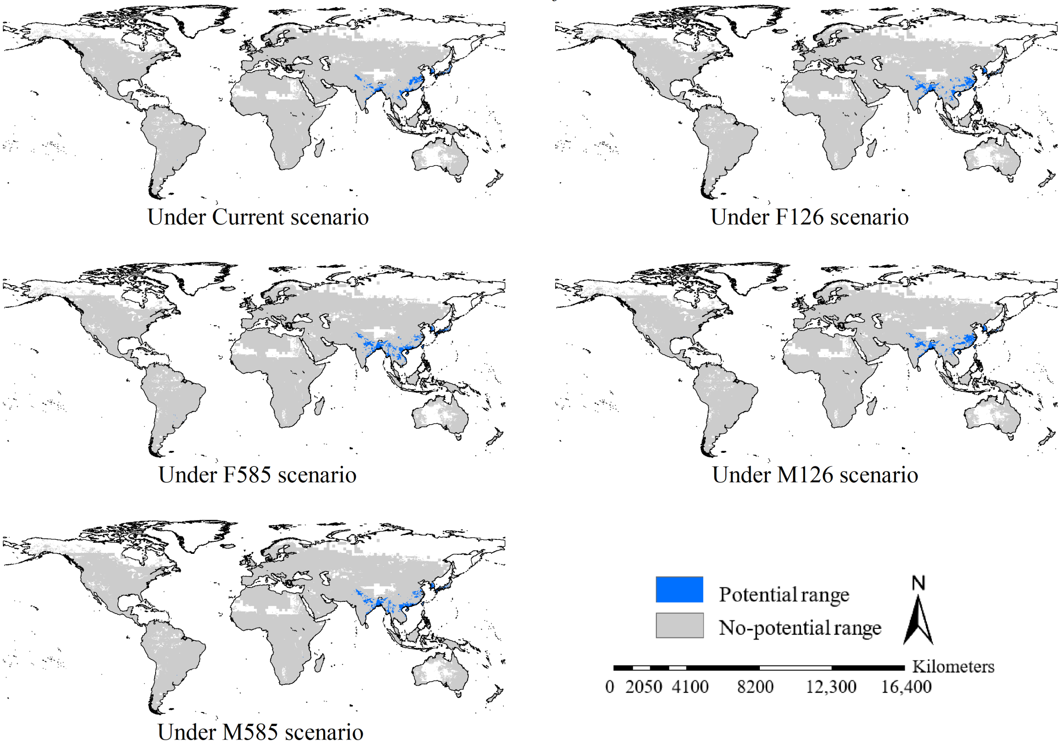

Sesamia nonagrioides

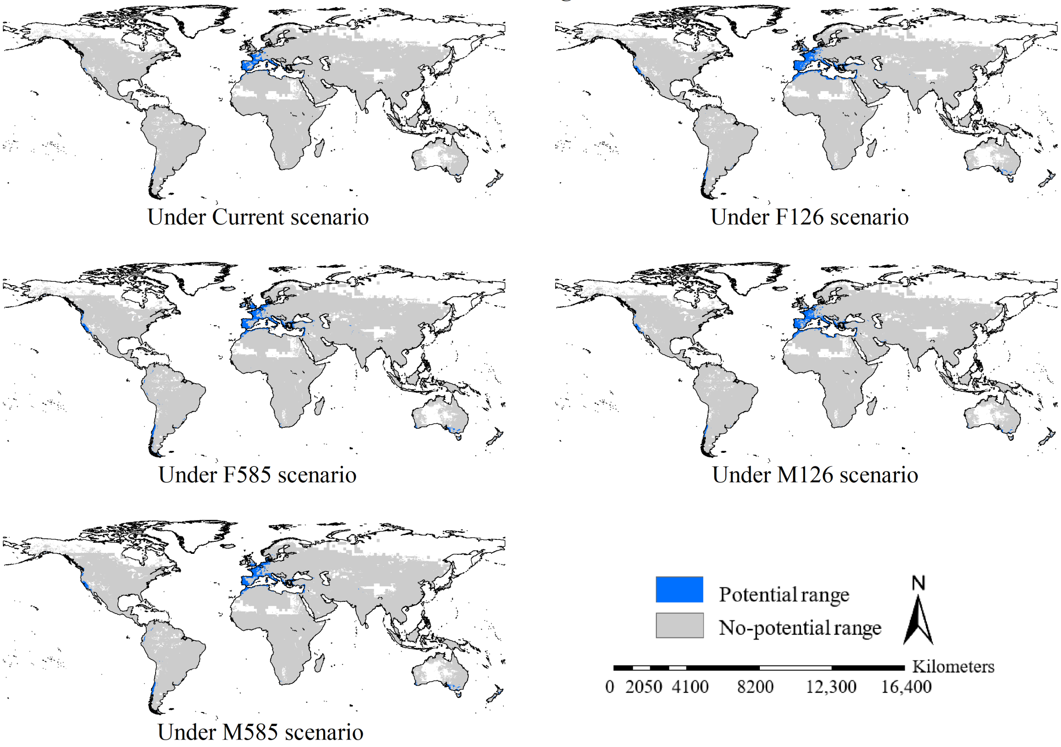

Sitophilus oryzae

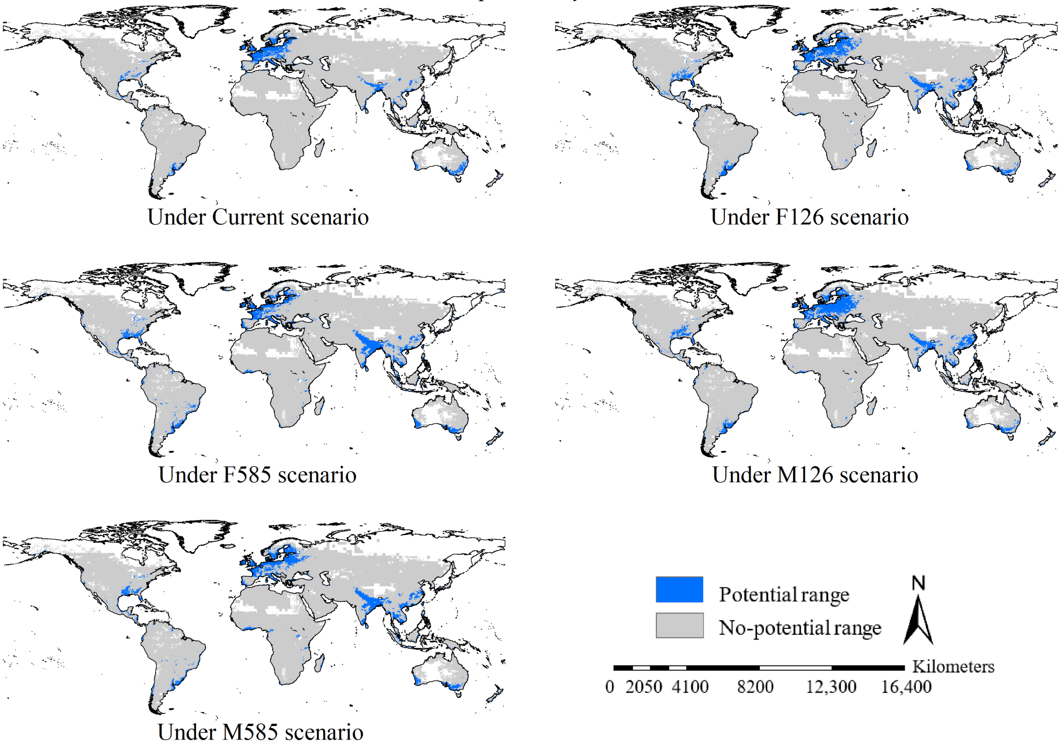

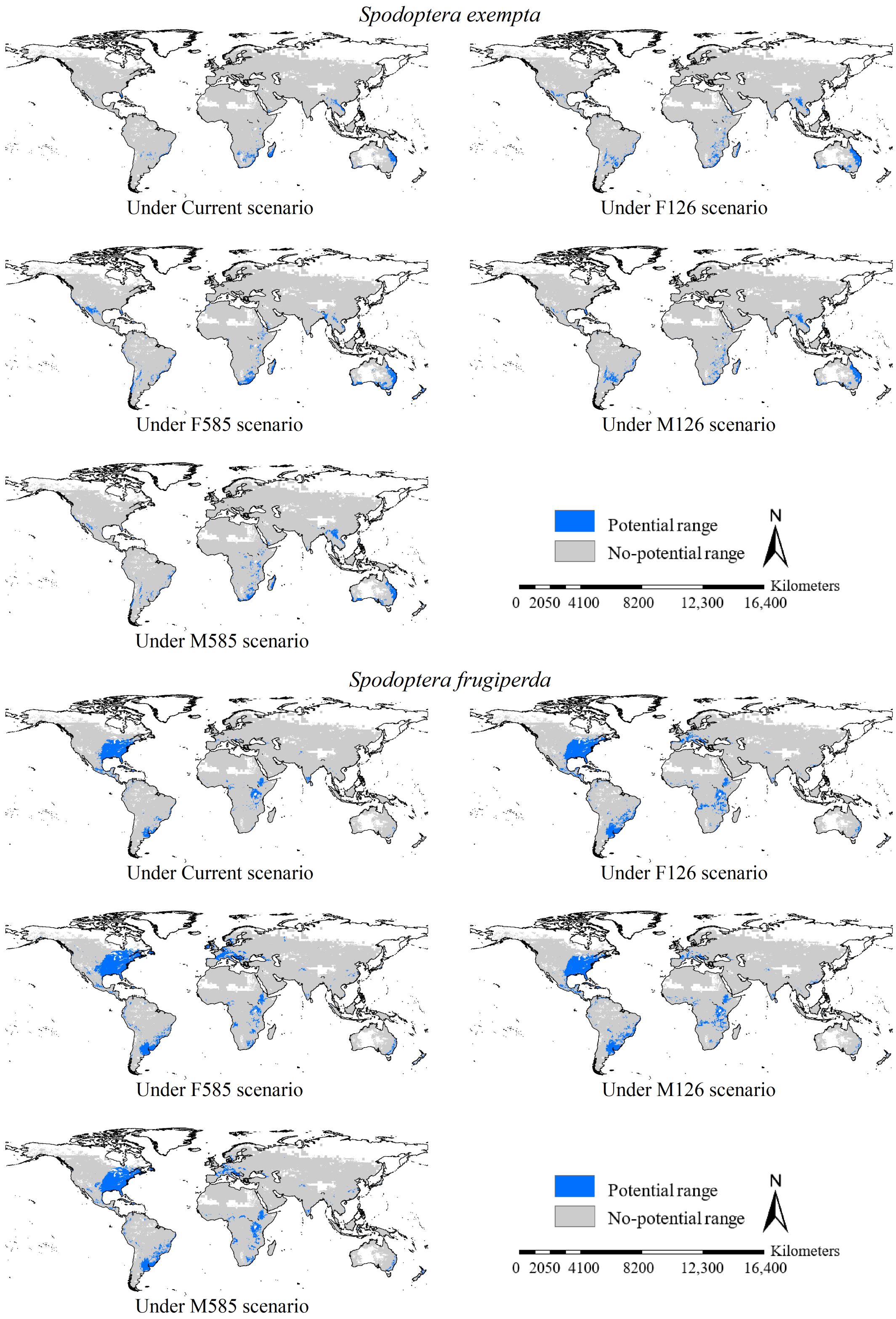
Spodoptera exempta
Under Current scenario
Under F126 scenario
Under F585 scenario
Under M126 scenario
Under M585 scenario
Potential range
No-potential range
N
Kilometers
0 2050 4100 8200 12,300 16,400
Spodoptera frugiperda
Under Current scenario
Under F126 scenario
Under F585 scenario
Under M126 scenario
Under M585 scenario
Potential range
No-potential range
N
Kilometers
0 2050 4100 8200 12,300 16,400
