# Supplementary material for: Future Range Shifts in Major Maize Insect Pests Suggest Their Increasing Impacts on Global Maize Production
Source: Insects. 2025 May 28;16(6):568. doi: 10.3390/insects16060568 (PMC12193563; doi:10.3390/insects16060568)
Supplement: Supplementary file 1 [file insects-16-00568-s001.zip › Table S1.pdf]

Table S1 The list of major insect pest species on maize

Busseola fusca
Chilo partellus
Chilo suppressalis
Cicadulina bipunctata
Cicadulina mbila
Dalbulus maidis
Diatraea grandiosella
Diatraea lineolata
Diatraea saccharalis
Diabrotica virgifera
Diabrotica virgifera virgifera
Elasmopalpus lignosellus
Eldana saccharina
Helicoverpa armigera
Ostrinia furnacalis
Ostrinia nubilalis
Rhopalosiphum maidis
Sesamia calamistis
Sesamia cretica
Sesamia inferens
Sesamia nonagrioides
Sitophilus oryzae
Spodoptera exempta
Spodoptera frugiperda
