# Supplementary material for: Future Range Shifts in Major Maize Insect Pests Suggest Their Increasing Impacts on Global Maize Production
Source: Insects. 2025 May 28;16(6):568. doi: 10.3390/insects16060568 (PMC12193563; doi:10.3390/insects16060568)
Supplement: Supplementary file 1 [file insects-16-00568-s001.zip › Table S4.pdf]

Table S4 Ten algorithms used to build preliminary models

We adopted the following ten algorithms in our SDMs:

Surface Range Envelope
Random Forest
Multiple Adaptive Regression Splines
XGBoost
Maximum Entropy Modeling
Artificial Neural Network
Generalized Linear Model
Classification Tree Analysis
Flexible Discriminant Analysis
Generalized Boosting Model
